# Age‐related alterations in human cortical microstructure across the lifespan: Insights from high‐gradient diffusion MRI

**DOI:** 10.1111/acel.14267

**Published:** 2024-08-08

**Authors:** Hansol Lee, Hong‐Hsi Lee, Yixin Ma, Laleh Eskandarian, Kyla Gaudet, Qiyuan Tian, Eva A. Krijnen, Andrew W. Russo, David H. Salat, Eric C. Klawiter, Susie Y. Huang

**Affiliations:** ^1^ Department of Radiology, Athinoula A. Martinos Center for Biomedical Imaging Massachusetts General Hospital Charlestown Massachusetts USA; ^2^ Department of Neurology Massachusetts General Hospital, Harvard Medical School Boston Massachusetts USA; ^3^ MS Center Amsterdam, Anatomy and Neurosciences, Amsterdam Neuroscience Amsterdam UMC Location VUmc Amsterdam The Netherlands; ^4^ Harvard‐MIT Division of Health Sciences and Technology Massachusetts Institute of Technology Cambridge Massachusetts USA

**Keywords:** aging, cell body, cortical microstructure, cortical volume, diffusion MRI, SANDI

## Abstract

The human brain undergoes age‐related microstructural alterations across the lifespan. Soma and Neurite Density Imaging (SANDI), a novel biophysical model of diffusion MRI, provides estimates of cell body (soma) radius and density, and neurite density in gray matter. The goal of this cross‐sectional study was to assess the sensitivity of high‐gradient diffusion MRI toward age‐related alterations in cortical microstructure across the adult lifespan using SANDI. Seventy‐two cognitively unimpaired healthy subjects (ages 19–85 years; 40 females) were scanned on the 3T Connectome MRI scanner with a maximum gradient strength of 300mT/m using a multi‐shell diffusion MRI protocol incorporating 8 *b*‐values and diffusion time of 19 ms. Intra‐soma signal fraction obtained from SANDI model‐fitting to the data was strongly correlated with age in all major cortical lobes (*r* = −0.69 to −0.60, FDR‐*p* < 0.001). Intra‐soma signal fraction (*r* = 0.48–0.63, FDR‐*p* < 0.001) and soma radius (*r* = 0.28–0.40, FDR‐*p* < 0.04) were significantly correlated with cortical volume in the prefrontal cortex, frontal, parietal, and temporal lobes. The strength of the relationship between SANDI metrics and age was greater than or comparable to the relationship between cortical volume and age across the cortical regions, particularly in the occipital lobe and anterior cingulate gyrus. In contrast to the SANDI metrics, all associations between diffusion tensor imaging (DTI) and diffusion kurtosis imaging metrics and age were low to moderate. These results suggest that high‐gradient diffusion MRI may be more sensitive to underlying substrates of neurodegeneration in the aging brain than DTI and traditional macroscopic measures of neurodegeneration such as cortical volume and thickness.

## Graphical abstract

We demonstrated age‐related alterations in gray matter microstructure in the cortex across the healthy adult lifespan using advanced biophysical modeling of diffusion MRI data acquired on the high‐gradient 3T Connectome MRI scanner. Lower intra‐soma signal fraction was observed in older adults compared to young and middle‐aged adults throughout the major cortical lobes. The regional cortical atrophy was accompanied by significant reductions in both intra‐soma signal fraction and soma radius.

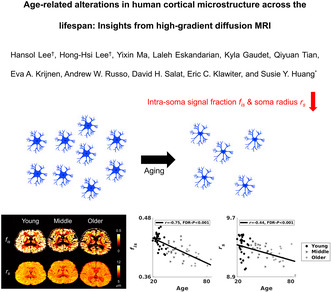

**Abbreviations**
AD: axial diffusivity
AK: axial kurtosis
ANCOVA: analysis of covariance
DKI: diffusion kurtosis imaging
DTI: diffusion tensor imaging
DWI: Diffusion‐weighted imaging
DW‐PGSE‐EPI: diffusion‐weighted pulsed‐gradient spin‐echo echo‐planar imaging
FA: fractional anisotropy
FDR: false discovery rate
FLAIR: fluid‐attenuated inversion recovery
GRAPPA: generalized autocalibrating partially parallel acquisition
MD: mean diffusivity
MEMPRAGE: multi‐echo magnetization‐prepared gradient echo
MK: mean kurtosis
MP‐PCA: Marchenko–Pastur principal component analysis
MRI: magnetic resonance imaging
NODDI: neurite orientation dispersion and density imaging
RD: radial diffusivity
RK: radial kurtosis
SANDI: soma and neurite density imaging
SMS: simultaneous multislice
SNR: signal‐to‐noise ratio
SMT: spherical mean technique
TE: echo time
TI: inversion time
TR: repetition time
WMTI: white matter tract integrity

## 1 INTRODUCTION

Age‐related alterations in brain structure are apparent at the microscopic scale on postmortem histopathology and at the macroscopic scale using in vivo magnetic resonance imaging (MRI) (Callaghan et al., 2014; Juraska & Lowry, 2012; Kemper, 1994; Liu et al., 2015; Raz, 2000; Salat, 2011; Sullivan & Pfefferbaum, 2006; Teissier et al., 2020). The gray matter undergoes gross morphometric changes with aging including thinning and volume loss of cortical, in particular gyral (Raz et al., 2010; Salat et al., 2004), and deep gray matter structures (Narvacan et al., 2017), which accelerate during the sixth and seventh decades (Kemper, 1994). Microscopically, histological studies have revealed widespread changes in dendritic structure, particularly in pyramidal neurons (Dickstein et al., 2007, 2013; Uylings & De Brabander, 2002), and reduction in cortical synapses with aging (Gibson, 1983; Masliah et al., 1993). Such alterations in brain tissue macro‐ and microstructure are associated with a decline in cognitive and sensorimotor function (Fleischman et al., 2015; Kohama et al., 2012). The onset of these tissue‐level changes and whether specific cortical regions are more vulnerable to age‐related degeneration remain unresolved. Noninvasive imaging markers that are sensitive to gray matter microstructural alterations prior to frank cortical volume loss are needed to shed light on the temporal evolution and regional specificity of microstructural changes underlying age‐related atrophy. Furthermore, maps of age‐associated gray matter microstructural changes would be invaluable for informing hypotheses concerning regionally specific processes driving age‐related atrophy and possibly differentiating normal aging from pathological neurodegeneration at an earlier stage.

Advances in neuroimaging techniques have paved the way toward evaluating macro‐ and microstructural changes in the human brain. High‐resolution anatomical *T*_1_‐weighted MRI has been applied to quantify morphological changes such as the loss of brain volume (Raz et al., 2010) and cortical thinning (Salat et al., 2004). Diffusion‐weighted imaging (DWI) measures water diffusion in brain tissue at the mesoscopic scale (Basser, 1995), allowing for the identification of microstructural alterations in white matter through its anisotropic organization. The application of conventional diffusion MRI models, such as diffusion tensor imaging (DTI) (Le Bihan et al., 2001), diffusion kurtosis imaging (DKI) (Jensen et al., 2005), neurite orientation dispersion and density imaging (NODDI) (Zhang et al., 2012), white matter tract integrity (WMTI) (Fieremans et al., 2013), axon diameter mapping (Alexander et al., 2010; Assaf et al., 2008; Fan et al., 2021; Huang et al., 2020), and multi‐compartmental spherical mean technique (SMT) (Kaden et al., 2016), have focused on examining alterations in white matter integrity in aging. Prior studies using diffusion MRI have shown age‐related changes in brain tissue microstructure, including increased mean diffusivity (MD) (Benitez et al., 2018; Madden et al., 2012), decreased fractional anisotropy (FA) (Benitez et al., 2018; Madden et al., 2012), decreased diffusional kurtosis (Benitez et al., 2018), and decreased neurite density (Merluzzi et al., 2016) in the white matter, possibly reflecting a combination of axonal damage and loss of myelin integrity. These changes have been observed in various neurodegenerative disorders, including Alzheimer's disease (Arab et al., 2018; Lo Buono et al., 2020), Parkinson's disease (Arab et al., 2018; Atkinson‐Clement et al., 2017; Wang et al., 2011; Zhang & Burock, 2020), and multiple sclerosis (Cercignani & Gandini Wheeler‐Kingshott, 2019; Sbardella et al., 2013). Additionally, previous studies have revealed a complex, nonlinear relationship between diffusion metrics such as FA, MD, and neurite density and age, with many prior groups demonstrating nonlinear U‐shaped curves of these diffusion metrics with age in the white matter, suggesting brain maturation followed by degeneration in later years (Beck et al., 2021; Burzynska et al., 2024; Kiely et al., 2022; Qian et al., 2020; Sullivan & Pfefferbaum, 2006; Westlye et al., 2010).

A major challenge in studying gray matter microstructure using diffusion MRI is that gray matter is not as orientationally coherent and heavily myelinated as white matter, resulting in an extremely low signal‐to‐noise ratio using strong diffusion‐weighting. While the association between gray matter diffusion metrics and age has not been extensively studied, the limited research on DTI in the cerebral cortex revealed a linear relationship of FA and MD with age, indicating an increase in both metrics with age (Benedetti et al., 2006; Ni et al., 2010; Rathi et al., 2014). Given that microstructural alterations in both white and gray matter occur with aging, it is necessary to develop and apply innovative diffusion MRI models that are sensitive to changes at this scale in the gray matter (Singh et al., 2024). Recently, the soma and neurite density imaging (SANDI) multi‐compartmental diffusion MRI model has been developed to estimate cell body (soma) radius and soma and neurite signal fractions in the brain gray matter (Palombo et al., 2020). SANDI moves beyond multi‐compartment models such as NODDI as it introduces a spherical cell body. As such, SANDI can be generalized to study brain tissue microstructure in both gray and white matter, offering potential insights into the histopathological underpinnings of age‐related microstructural alterations throughout the brain. The estimated cell body and neurite signal fractions from the SANDI model have shown good agreement with cytoarchitectural and myeloarchitectural features across different brain regions, as reflected in histological analyses (Ianuş et al., 2022; Palombo et al., 2020). The advent of more powerful gradient systems on commercially available scanners is making the SANDI approach feasible for clinical research in the near future (Fan et al., 2022; Foo et al., 2020; Huang et al., 2021; Vachha & Huang, 2021). The feasibility of using the SANDI model with lower performance gradients has been shown, demonstrating that SANDI can be a practical method for in vivo characterization of brain tissues using clinical scanners (Palombo et al., 2020; Schiavi et al., 2023). In addition, SANDI has been applied to study the microstructural substrate of gray matter degeneration in neurological diseases such as multiple sclerosis, further highlighting its potential for detecting and monitoring neurodegeneration in normal aging and multiple sclerosis (Krijnen et al., 2023, 2024; Margoni et al., 2023; Schiavi et al., 2023).

The goal of this study was to investigate the associations between cortical gray matter micro‐ and macrostructure, and age in healthy individuals across the lifespan using high‐gradient diffusion MRI. We applied the SANDI model to multi‐shell diffusion MRI data obtained on the 3T Connectome MRI scanner. We postulated that within cortical gray matter, intra‐soma signal fraction and apparent soma radius would decrease with age corresponding to cortical atrophy. We also considered the possibility of complex, nonlinear associations between gray matter microstructural metrics with age, given the relationship between white matter microstructural metrics and age reported in previous studies using diffusion MRI. We also hypothesized that intra‐soma signal fraction would be more strongly correlated with age than cortical volume, and that the SANDI model would be more sensitive to age‐related changes in cortical tissue microstructure than other conventional diffusion MRI models, specifically DTI and DKI.

## 2 RESULTS

### 2.1 Participant demographics and age‐related alterations in cortical macrostructure (cortical volume)

**FIGURE 1:**
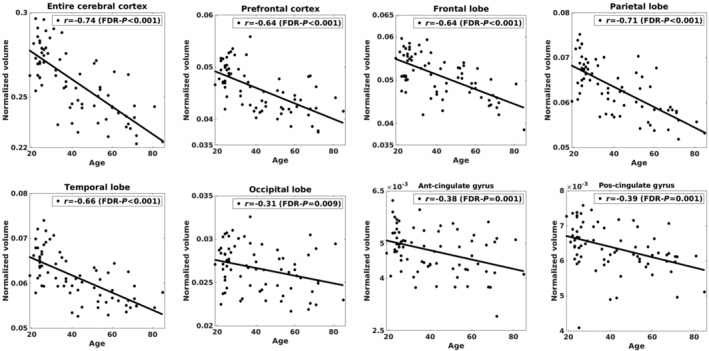
Correlations between age and normalized cortical volume in the cerebral cortex and each major cortical region. *r* value is Pearson's correlation coefficient with *p*‐value after correcting for multiple comparisons using the FDR.

Table 1 shows the characteristics of the included participants. The participants were divided into three age groups, whose age threshold was determined to represent different life phases across the lifespan (Lachman, 2004): a young adult group (under 34 years), a middle‐aged group (35–54 years), and an older adult group (over 55 years). The young adult group consisted of 33 participants (25.5 ± 3.6 years, 18 female). The middle‐aged group included 21 participants (44.3 ± 6.6 years, 13 female). The older adult group comprised 18 subjects (67.1 ± 7.7 years, 9 female). The entire cerebral cortex exhibited a negative correlation between age and normalized cortical volume (*r* = −0.74, FDR‐*p* < 0.001), which was estimated from a *T*_1_‐weighted anatomical image (Figure 1). Among the major cortical regions, the prefrontal cortex, frontal, parietal, and temporal lobes displayed a strong association between age and cortical volume (*r* = −0.71 to −0.64, FDR‐*p* < 0.001). The occipital lobe, anterior cingulate gyrus, and posterior cingulate gyrus showed a comparatively lower but still significant correlation (*r* = −0.39 to −0.31, FDR‐*p* = 0.001–0.009). Within the eight cortical subregions, the lateral prefrontal cortex, orbital frontal cortex, inferior parietal cortex, inferior temporal cortex, and hippocampus revealed a negative correlation between age and cortical volume (*r* = −0.68 to −0.37, FDR‐*p* < 0.002) (Figure S1). The fusiform cortex showed a modest yet significant correlation between age and cortical volume (*r* = −0.25, FDR‐*p* = 0.048). The entorhinal cortex and primary visual cortex did not show significant correlations between age and cortical volume.

**TABLE 1:** Demographic characteristics of the participants.

Group	Sample size		Age	
	Number	Sex	Mean ± SD (years)	Range (years)
Young	33	18 F/15 M	25.5 ± 3.6	19–34
Middle	21	13 F/8 M	44.3 ± 6.6	35–54
Older	18	9 F/9 M	67.1 ± 7.7	55–85
Total	72	40 F/32 M	41.4 ± 17.9	19–85

### 2.2 SANDI results

**FIGURE 2:**
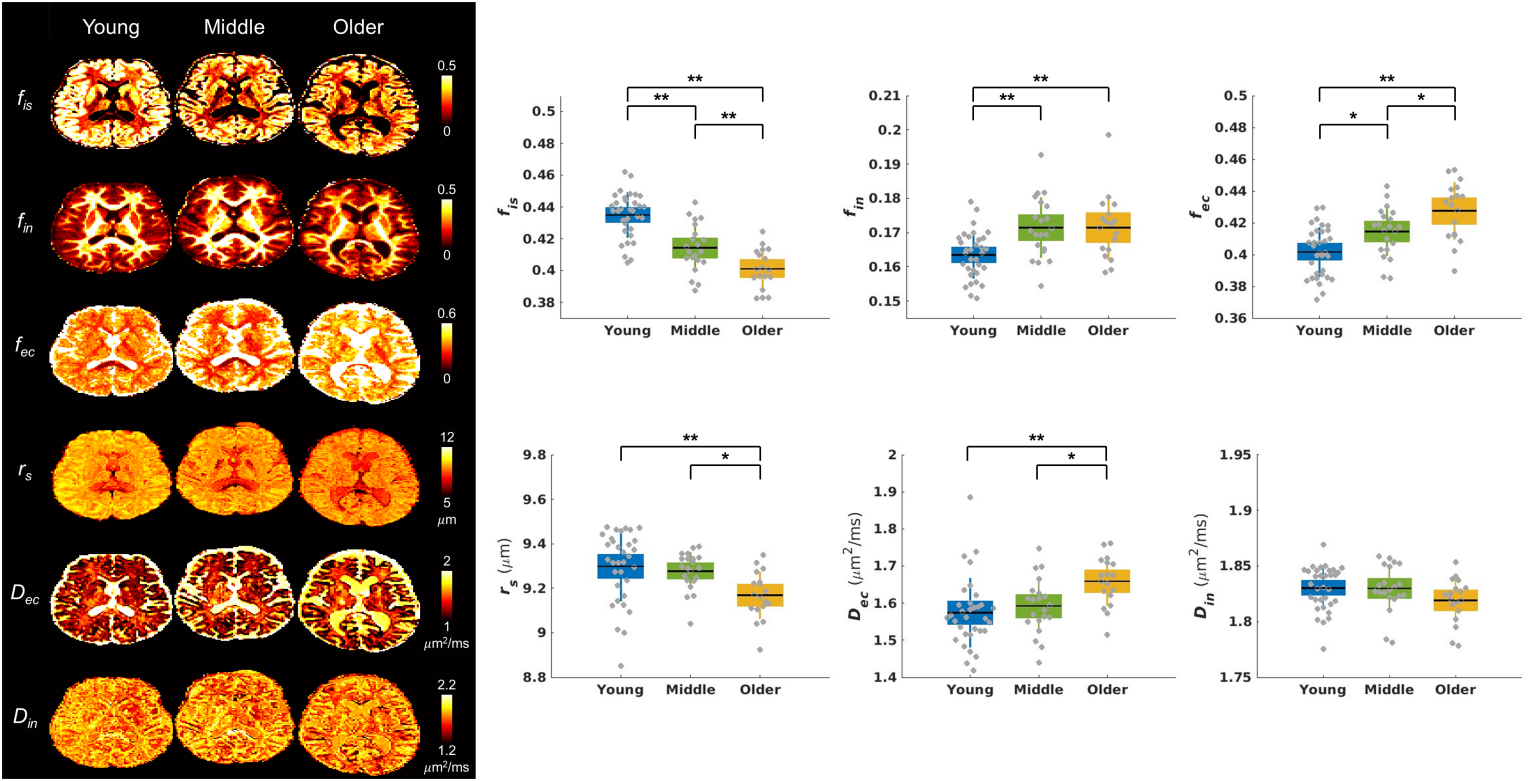
Representative maps of SANDI metrics and the distribution of SANDI metrics averaged across the cerebral cortex for each group. *: *p* < 0.05 and **: *p* < 0.005 in an unpaired *t*‐test adjusting for multiple comparisons using the FDR.

Figure 2 shows SANDI metric maps, such as the intra‐soma signal fraction *f*_is_, intra‐neurite signal fraction *f*_in_, extracellular signal fraction *f*_ec_, soma radius *r*_s_, extracellular diffusivity *D*_ec_, and intra‐neurite diffusivity *D*_in_, for representative individuals in the young, middle‐aged, and older adult groups.

### 2.3 Age‐related alterations in SANDI microstructural metrics

Comparing SANDI metrics in the entire cerebral cortex among three age groups (Figure 2), the intra‐soma signal fraction *f*_is_ was lower in the older adults compared to the two other groups (FDR‐*p* < 0.001 for both comparisons) (Table S1). The intra‐soma signal fraction *f*_is_ was also lower in the middle‐aged adults compared to the young adults (FDR‐*p* < 0.001). The intra‐neurite signal fraction *f*_in_ was higher in the middle‐aged adults (FDR‐*p* = 0.003) and older adults (FDR‐*p* = 0.002) compared to the young adults. The extracellular signal fraction *f*_ec_ was significantly higher in the older adults compared to the young (FDR‐*p* < 0.001) and middle‐aged adults (FDR‐*p* = 0.02). Similarly, the extracellular signal fraction *f*_ec_ was higher in the middle‐aged adults compared to the young adults (FDR‐*p* = 0.01). The soma radius *r*_s_ in the older adults was significantly smaller than in the young adults (FDR‐*p* = 0.003) and middle‐aged adults (FDR‐*p* = 0.02). The extracellular diffusivity *D*_ec_ was higher in the older adult group compared to the young adult group (FDR‐*p* = 0.003) and middle‐aged adults (FDR‐*p* = 0.02). The intra‐neurite diffusivity *D*_in_ showed no significant differences among the three age groups. Table S2 summarizes the SANDI metrics in cerebral white matter and major cortical regions averaged over all participants.

**FIGURE 3:**
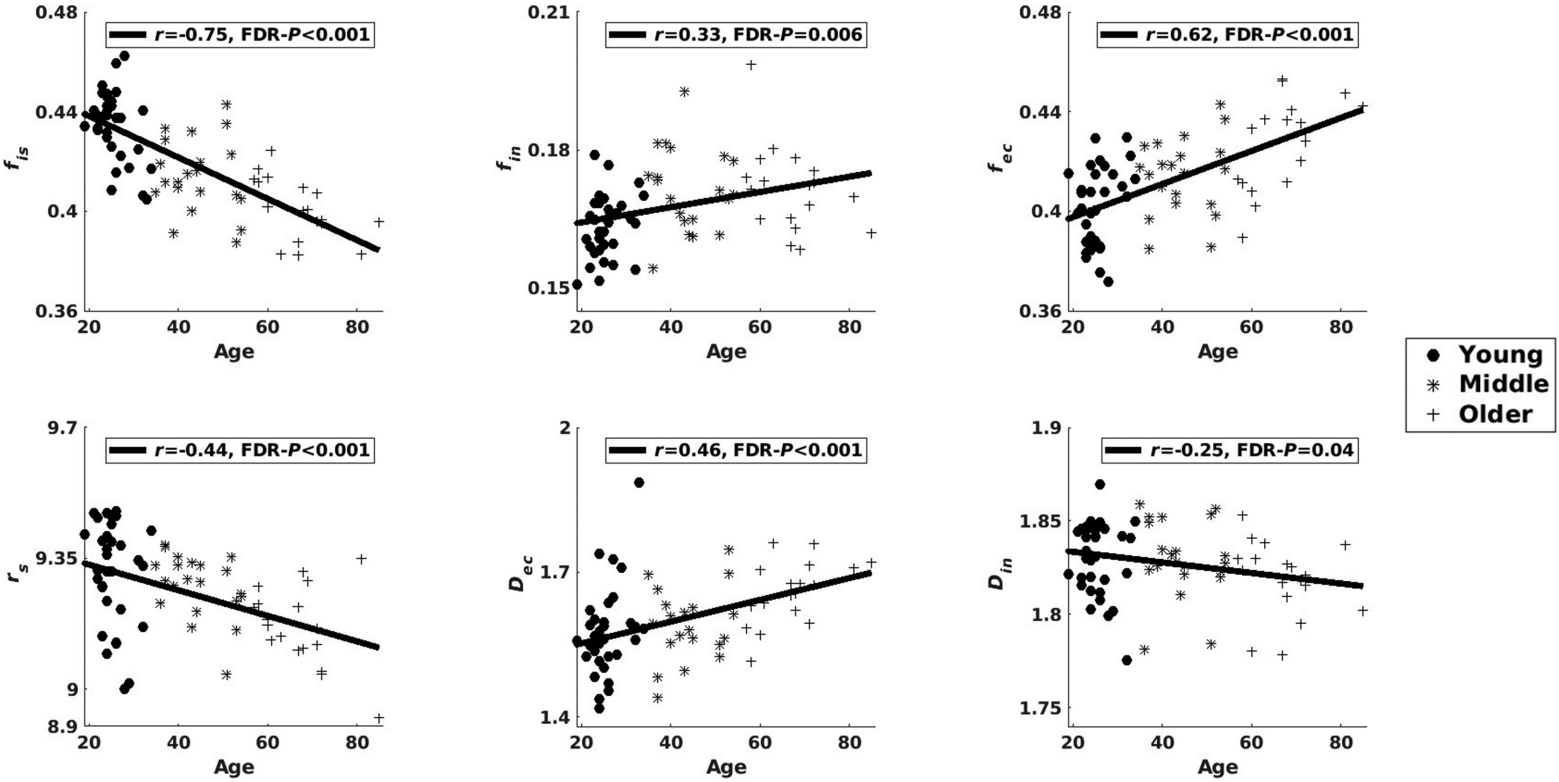
Correlations between age and SANDI metrics in the entire cerebral cortex. *r* value is Pearson’s correlation coefficient with *P*‐value after correcting for multiple comparisons using the FDR. The unit for *r_s_* is μm. The units for *D_ec_* and *D_in_* are μm^2^/ms.

Evaluating the relationship between age and gray matter microstructure, the scatter plots of the SANDI metrics versus age in the entire cerebral cortex (Figure 3) showed that the intra‐soma signal fraction *f*_is_ (*r* = −0.75, FDR‐*p* < 0.001), soma radius *r*_s_ (*r* = −0.44, FDR‐*p* < 0.001), intra‐neurite diffusivity *D*_in_ (*r* = −0.25, FDR‐*p* = 0.04) decreased with age, whereas the intra‐neurite signal fraction *f*_in_ (*r* = 0.33, FDR‐*p* = 0.006), extracellular signal fraction *f*_ec_ (*r* = 0.62, FDR‐*p* < 0.001), and extracellular diffusivity *D*_ec_ (*r* = 0.46, FDR‐*p* < 0.001) increased with age.

**FIGURE 4:**
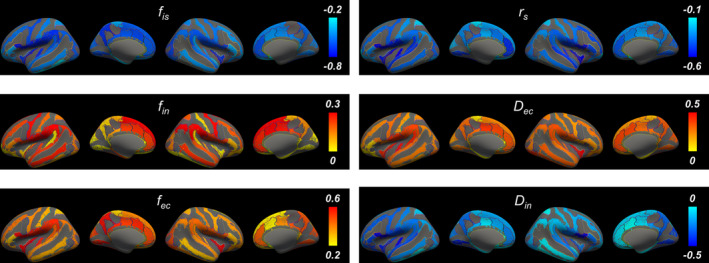
Correlations between age and the SANDI metrics on the inflated cortical surface. The correlations are exclusively depicted for each individual brain gyrus.

The correlations observed between SANDI metrics and age were consistent across the major cortical regions (Figure 4 and Table 2). Exceptions to this trend were either the absence of significant correlations or weaker significance between intra‐neurite signal fraction *f*_in_ and age, as well as intra‐neurite diffusivity *D*_in_ in certain lobes. Additionally, the intra‐soma signal fraction *f*_is_ showed the strongest correlation with age across all major cortical regions (all FDR‐*p* < 0.001) compared to the other SANDI metrics.

**TABLE 2:** Correlations between SANDI metrics and age and between SANDI metrics and the normalized cortical volume in the major cortical regions.

Age								
	Entire cerebral cortex	Prefrontal	Frontal	Parietal	Temporal	Occipital	Anterior cingulate	Posterior cingulate
*f*_is_	**−0.75**	**−0.64**	**−0.65**	**−0.68**	**−0.61**	**−0.69**	**−0.66**	**−0.60**
	(<0.001*)	(<0.001*)	(<0.001*)	(<0.001*)	(<0.001*)	(<0.001*)	(<0.001*)	(<0.001*)
	(<0.001*)	(<0.001*)	(<0.001*)	(<0.001*)	(<0.001*)	(<0.001*)	(<0.001*)	(<0.001*)
*f*_in_	**0.33**	0.23	**0.43**	0.24	0.23	−0.08	**0.28**	0.23
	(0.005*)	(0.0497*)	(<0.001*)	(0.04*)	(0.05)	(0.49)	(0.02*)	(0.06)
	(0.008*)	(0.06)	(<0.001*)	(0.05)	(0.06)	(0.49)	(0.02*)	(0.06)
*f*_ec_	**0.62**	**0.58**	**0.44**	**0.53**	**0.52**	**0.60**	**0.53**	**0.57**
	(<0.001*)	(<0.001*)	(<0.001*)	(<0.001*)	(<0.001*)	(<0.001*)	(<0.001*)	(<0.001*)
	(<0.001*)	(<0.001*)	(<0.001*)	(<0.001*)	(<0.001*)	(<0.001*)	(<0.001*)	(<0.001*)
*r*_s_ (μm)	**−0.44**	**−0.45**	**−0.33**	**−0.35**	**−0.41**	**−0.48**	**−0.58**	**−0.40**
	(<0.001*)	(<0.001*)	(0.005*)	(0.003*)	(<0.001*)	(<0.001*)	(<0.001*)	(<0.001*)
	(<0.001*)	(<0.001*)	(0.008*)	(0.005*)	(<0.001*)	(<0.001*)	(<0.001*)	(<0.001*)
*D*_ec_ (μm^2^/ms)	**0.46**	**0.34**	**0.31**	**0.37**	**0.40**	**0.37**	**0.44**	**0.34**
	(<0.001*)	(0.003*)	(0.008*)	(0.001*)	(<0.001*)	(0.001*)	(<0.001*)	(0.003*)
	(<0.001*)	(0.005*)	(0.01*)	(0.002*)	(<0.001*)	(0.002*)	(<0.001*)	(0.005*)
*D*_in_ (μm^2^/ms)	**−0.25**	**−0.26**	−0.13	−0.18	−0.16	**−0.37**	−0.24	−0.22
	(0.04*)	(0.03*)	(0.28)	(0.12)	(0.18)	(0.002*)	(0.0453*)	(0.06)
	(0.0456*)	(0.04*)	(0.49)	(0.13)	(0.19)	(0.003*)	(0.05)	(0.07)

Normalized cortical volume								
*f*_is_	**0.74**	**0.58**	**0.48**	**0.63**	**0.51**	0.26	**0.29**	0.19
	(<0.001*)	(<0.001*)	(<0.001*)	(<0.001*)	(<0.001*)	(0.03*)	(0.01*)	(0.12)
	(<0.001*)	(<0.001*)	(<0.001*)	(<0.001*)	(<0.001*)	(0.06)	(0.04*)	(0.16)
*f*_in_	**−0.36**	−0.23	**−0.28**	−0.20	−0.26	0.07	**−0.32**	−0.13
	(0.003*)	(0.06)	(0.02*)	(0.10)	(0.03*)	(0.58)	(0.007*)	(0.39)
	(0.009*)	(0.09)	(0.04*)	(0.15)	(0.06)	(0.60)	(0.02*)	(0.45)
*f*_ec_	**−0.60**	**−0.52**	**−0.34**	**−0.51**	**−0.39**	−0.24	−0.10	−0.15
	(<0.001*)	(<0.001*)	(0.004*)	(<0.001*)	(<0.001*)	(0.047*)	(0.40)	(0.20)
	(<0.001*)	(<0.001*)	(0.01*)	(<0.001*)	(0.003*)	(0.08)	(0.46)	(0.25)
*r*_s_ (μm)	**0.41**	**0.38**	**0.40**	**0.37**	**0.28**	0.20	**0.28**	0.12
	(<0.001*)	(<0.001*)	(<0.001*)	(0.001*)	(0.02*)	(0.09)	(0.02*)	(0.30)
	(0.002*)	(0.003*)	(0.002*)	(0.005*)	(0.04*)	(0.13)	(0.04*)	(0.37)
*D*_ec_ (μm^2^/ms)	**−0.42**	−0.23	−0.25	**−0.28**	−0.16	−0.11	−0.09	−0.09
	(<0.001*)	(0.05)	(0.04*)	(0.02*)	(0.18)	(0.35)	(0.44)	(0.46)
	(0.002*)	(0.08)	(0.07)	(0.04*)	(0.23)	(0.41)	(0.48)	(0.49)
*D*_in_ (μm^2^/ms)	0.23	**0.28**	0.17	**0.29**	0.06	0.19	0.10	0.04
	(0.06)	(0.02*)	(0.16)	(0.01*)	(0.60)	(0.11)	(0.42)	(0.72)
	(0.09)	(0.04*)	(0.21)	(0.04*)	(0.61)	(0.16)	(0.47)	(0.72)

*Not*e: Data are reported as Pearson's correlation coefficient with corresponding uncorrected *p*‐values (first pair of parentheses) followed by FDR‐corrected *p*‐values (second pair of parentheses). Numbers in bold indicate results that are statistically significant after FDR correction. Values with the asterisk denotes statistical significance (*p* < 0.05 or FDR‐*p* < 0.05) in Pearson's correlation coefficient analysis.

In the eight cortical subregions, the strongest relationships between age and intra‐soma signal fraction *f*_is_ were identified in the lateral prefrontal cortex, orbital frontal cortex, inferior parietal cortex, fusiform cortex, and primary visual cortex (*r* = −0.65 to −0.57, FDR‐*p* < 0.001) (Table S3). Lower yet significant correlations between age and intra‐soma signal fraction were observed in the inferior temporal cortex (*r* = −0.35, FDR‐*p* = 0.007) and hippocampus (*r* = −0.46, FDR‐*p* < 0.001). The entorhinal cortex showed no correlation between age and any of the SANDI metrics.

### 2.4 Relationships between SANDI metrics and cortical volume

**FIGURE 5:**
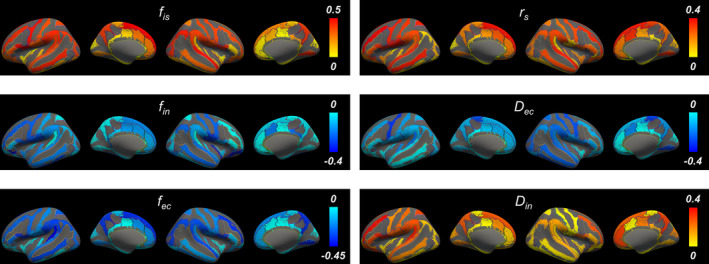
Correlations between the normalized volume and the SANDI metrics on the inflated cortical surface. The correlations are exclusively depicted for each individual brain gyrus.

To evaluate the relationship between gray matter microstructure and macrostructure, we studied the correlation between SANDI metrics and normalized cortical volume. Of all the SANDI parameters studied, in the entire cerebral cortex, the intra‐soma signal fraction *f*_is_ (*r* = 0.74, FDR‐*p* < 0.001) showed the most significant correlation with overall normalized cortical volume (Figure 5 and Table 2). The soma radius *r*_s_ (*r* = 0.41, FDR‐*p* = 0.002) was also positively correlated with normalized cortical volume. The intra‐neurite signal fraction *f*_in_ (*r* = −0.36, FDR‐*p* = 0.009), extracellular signal fraction *f*_ec_ (*r* = −0.60, FDR‐*p* < 0.001), and extracellular diffusivity *D*_ec_ (*r* = −0.42, FDR‐*p* = 0.002) were inversely correlated with normalized cortical volume.

In the major cortical regions, the intra‐soma signal fraction *f*_is_ was positively correlated with normalized cortical volume in most of the major cortical lobes (*r* = 0.29–0.63, FDR‐*p* < 0.04) except for the occipital lobe and posterior cingulate gyrus (Table 2). The intra‐neurite signal fraction *f*_in_ was negatively correlated with normalized cortical volume in the frontal lobe (*r* = −0.28, FDR‐*p* = 0.04) and anterior cingulate gyrus (*r* = −0.32, FDR‐*p* = 0.02). Moreover, the extracellular signal fraction *f*_ec_ was negatively correlated with normalized cortical volume in the prefrontal cortex (*r* = −0.52, FDR‐*p* < 0.001), frontal (*r* = −0.34, FDR‐*p* = 0.01), parietal (*r* = −0.51, FDR‐*p* < 0.001), and temporal lobes (*r* = −0.39, FDR‐*p* = 0.003). Soma radius *r*_s_ was positively correlated with normalized cortical volume in the prefrontal region (*r* = 0.38, FDR‐*p* = 0.003), frontal (*r* = 0.40, FDR‐*p* = 0.002), parietal (*r* = 0.37, FDR‐*p* = 0.005), and temporal lobes (*r* = 0.28, FDR‐*p* = 0.04), and anterior cingulate gyrus (*r* = 0.28, FDR‐*p* = 0.04). The extracellular diffusivity *D*_ec_ was negatively correlated with normalized cortical volume only in the parietal lobes (*r* = −0.28, FDR‐*p* = 0.04). The intra‐neurite diffusivity *D*_in_ was positively associated with normalized cortical volume in the prefrontal region (*r* = 0.28, FDR‐*p* = 0.04) and parietal lobe (*r* = 0.29, FDR‐*p* = 0.04).

Within the eight cortical subregions (Table S4), the lateral prefrontal cortex indicated a positive correlation of intra‐soma signal fraction *f*_is_ (*r* = 0.62, FDR‐*p* < 0.001), soma radius *r*_s_ (*r* = 0.41, FDR‐*p* = 0.004), and intra‐neurite diffusivity *D*_in_ (*r* = 0.37, FDR‐*p* = 0.01) in relation to the normalized cortical volume, whereas there was a negative association for the extracellular signal fraction *f*_ec_ (*r* = −0.52, FDR‐*p* < 0.001). The inferior parietal cortex displayed a significant correlation of intra‐soma signal fraction *f*_is_ (*r* = 0.52, FDR‐*p* < 0.001) and extracellular signal fraction *f*_ec_ (*r* = −0.42, FDR‐*p* = 0.004) with respect to the normalized cortical volume. However, the other six regions did not exhibit any correlation between the macro‐ and microstructure.

### 2.5 Differences between alterations in cortical microstructure and macrostructure with aging

The correlations between age, normalized cortical volume, and intra‐soma signal fraction *f*_is_ are illustrated in Figures S2 and S3. In the major cortical regions, the alterations in microstructure and macrostructure associated with aging were similar in the prefrontal cortex, frontal, parietal, and temporal lobes, and posterior cingulate gyrus. However, the occipital lobe (*z*‐score = 3.10; *p* = 0.002) and anterior cingulate gyrus (*z*‐score = 2.31; *p* = 0.02) showed a stronger correlation between age and intra‐soma signal fraction *f*_is_ compared to the correlation between age and normalized cortical volume. This pattern remained consistent in specific cortical subregions. There was a significant difference between the alterations in cortical microstructure and macrostructure related to aging in the fusiform cortex (*z*‐score = 2.30; *p* = 0.02) and primary visual cortex (*z*‐score = 3.86; *p* < 0.001).

### 2.6 The influence of free water signals on the correlations

The free water signal fraction *f*_iso_ derived from NODDI exhibited a positive correlation with age across all brain regions (*r* = 0.25 to 0.65, FDR‐*p* < 0.047) (Table S5). It showed a negative correlation with the normalized volume of brain regions (*r* = −0.61 to 0.31, FDR‐*p* < 0.01), except for the occipital lobe, anterior cingulate, and posterior cingulate. When free water signal fraction *f*_iso_ was added as an additional covariate in the correlation coefficient analysis, the correlation coefficients for extracellular signal fraction *f*_ec_ and extracellular diffusivity *D*_ec_ with age and normalized volume generally decreased, and significant relationships disappeared in most cortical regions (Tables S6 and S7). However, the relationships of intra‐soma signal fraction *f*_is_ with age and normalized volume remain significant for most of the cortical lobes.

### 2.7 SANDI metrics in the deep gray matter

In the caudate and putamen, all SANDI metrics except intra‐neurite diffusivity *D*_in_ showed significant associations with age. There was a negative correlation between age and intra‐soma signal fraction *f*_is_ (*r* = −0.65, FDR‐*p* < 0.001 and *r* = −0.58, FDR‐*p* < 0.001, respectively) (Table S8). The pallidum and thalamus did not exhibit a significant correlation of intra‐soma signal fraction *f*_is_ with age, but both showed a negative correlation between soma radius *r*_s_ with age (*r* = −0.32, FDR‐*p* = 0.01 for both regions). The caudate and putamen indicated a positive correlation of intra‐soma signal fraction *f*_is_ (*r* = 0.39, FDR‐*p* = 0.003 and *r* = 0.41, FDR‐*p* = 0.002, respectively) and soma radius *r*_s_ (*r* = 0.55, FDR‐*p* < 0.001 and *r* = 0.56, FDR‐*p* < 0.001, respectively) in relation to the normalized cortical volume, whereas there was a negative association for the extracellular signal fraction extracellular diffusivity *D*_ec_ (*r* = −0.33, FDR‐*p* = 0.01 and *r* = −0.36, FDR‐*p* = 0.007, respectively) (Table S9).

### 2.8 SANDI metrics in the cerebral white matter

The cerebral white matter showed a negative relationship between age and normalized volume (*r* = −0.28, *p* = 0.02) (Figure S4). None of the SANDI metrics were significantly associated with age in the cerebral white matter (Table S10). However, significant correlations were observed between the normalized volume of cerebral white matter and several SANDI metrics: the intra‐neurite signal fraction *f*_in_ (*r* = 0.51, FDR‐*p* < 0.001), extracellular signal fraction *f*_ec_ (*r* = −0.32, FDR‐*p* = 0.02), soma radius *r*_s_ (*r* = 0.48, FDR‐*p* < 0.001) and intra‐neurite diffusivity *D*_in_ (*r* = 0.63, FDR‐*p* < 0.001).

### 2.9 Relationships between DTI/DKI metrics and age

In the cerebral white matter, in keeping with previous reports, FA showed a negative correlation with age (*r* = −0.50, FDR‐*p* < 0.001), whereas radial diffusivity (RD) exhibited a positive correlation with age (*r* = 0.37, FDR‐*p* = 0.004) (Table S11). In all the major cortical regions, all DTI metrics (FA, axial diffusivity [AD], RD, and MD) significantly increased with age (*r* = 0.34–0.47, FDR‐*p* < 0.009). FA was positively correlated with age in the frontal (*r* = 0.46, FDR‐*p* < 0.001) and parietal lobes (*r* = 0.47, FDR‐*p* < 0.001). MD exhibited a positive correlation with age in most of the major cortical regions (*r* = 0.26–0.43, FDR‐*p* < 0.04) except for the frontal lobe. Furthermore, kurtosis values (axial kurtosis [AK], radial kurtosis [RK], and mean kurtosis [MK]) showed positive age‐related correlations in most of the major cortical lobes, except the occipital lobe (Table S12). In contrast to the SANDI metrics, all associations between DTI and DKI metrics and age were low to moderate.

### 2.10 Relationships between DTI/DKI metrics and cortical volume

The DTI metrics did not exhibit any significant correlation with the normalized cortical volume (Table S13). The DKI metrics displayed negative correlations with the normalized volume in the overall cortex (*r* = −0.44, FDR‐*p* = 0.001 for AK; *r* = −0.28, FDR‐*p* = 0.03 for RK; *r* = −0.42, FDR‐*p* = 0.005 for MK) (Table S14). All DKI metrics were also correlated with the normalized volume in the prefrontal cortex (*r* = −0.44 to −0.31, FDR‐*p* < 0.03). Specifically, AK showed an additional negative correlation with the normalized cortical volume in the frontal (*r* = −0.30, FDR‐*p* = 0.03), parietal (*r* = −0.27, FDR‐*p* = 0.04), and temporal lobes (*r* = −0.44, FDR‐*p* = 0.001), anterior cingulate gyrus (*r* = −0.38, FDR‐*p* = 0.006), and posterior cingulate gyrus (*r* = −0.28, FDR‐*p* = 0.03). MK similarly showed a negative correlation with normalized cortical volume in the parietal (*r* = −0.29, FDR‐*p* = 0.03) and temporal lobes (*r* = −0.35, FDR‐*p* = 0.01) and anterior cingulate gyrus (*r* = −0.29, FDR‐*p* = 0.03).

### 2.11 Nonlinear trends of diffusion metrics with age

In the cerebral white matter, a quadratic regression curve was applied to all DTI and DKI metrics, except for FA, in relation to age (Figures S5 and S6). Meanwhile, in the cerebral cortex, only FA, AK, and RK displayed a quadratic trend. For SANDI metrics, the intra‐neurite signal fraction *f*_in_ and intra‐neurite diffusivity *D*_in_ exhibited inverted U‐shaped patterns in the cerebral cortex, with peak ages at 55 and 30 years, respectively (Figure S7). Quadratic regression (*R* ^2^ = 0.233) provided a better fit for intra‐neurite signal fraction *f*_in_ than linear regression (*R* ^2^ = 0.149), while other SANDI metrics had comparable fitting qualities with both methods (Table S17). Similarly, in the cerebral white matter, the intra‐neurite signal fraction *f*_in_ and intra‐neurite diffusivity *D*_in_, followed inverted *U*‐shaped trends, whereas the intra‐soma signal fraction *f*_is_, extracellular signal fraction *f*_ec_, and extracellular diffusivity *D*_ec_ exhibited *U*‐shaped patterns. The *R* ^2^ value for quadratic regression of intra‐neurite signal fraction *f*_in_ was 0.265, compared to 0.203 for linear regression.

## 3 DISCUSSION

In this work, we applied SANDI, a recently developed multi‐compartmental diffusion MRI model for gray matter, to investigate age‐related microstructural alterations in the cortex of healthy adults across the lifespan who were scanned on the high‐gradient 3T Connectome MRI system. We observed significantly lower intra‐soma signal fraction *f*_is_ and soma radius *r*_s_ in older adults compared to young and middle‐aged adults. The intra‐soma signal fraction *f*_is_ and soma radius *r*_s_ also showed a significant negative correlation with age across the major cortical lobes and were accompanied by higher extracellular signal fraction *f*_ec_ and extracellular diffusivity *D*_ec_ in older adults. The relationship between microstructure (intra‐soma signal fraction *f*_is_) and macrostructure (cortical volume) was more pronounced in the prefrontal cortex, frontal, parietal, and temporal lobes than the occipital lobe, anterior cingulate gyrus, and posterior cingulate gyrus. Furthermore, the strengths of the associations between the SANDI microstructural metrics and age were greater than or comparable to the associations between cortical volume and age in many cortical regions, particularly in the occipital lobe and anterior cingulate gyrus. Of all the diffusion MRI measures studied here, the intra‐soma signal fraction *f*_is_ from the SANDI model showed the strongest and most significant associations with age and normalized cortical volume in the cortical gray matter. We also demonstrated that the SANDI model is more robust and applicable to observe the age‐related microstructural changes in the gray matter compared to white matter, aligning with the originality of the SANDI approach in reflecting soma‐related information. These findings suggest that the SANDI model applied to high‐gradient diffusion MRI data may provide noninvasive imaging markers of cortical microstructural alterations related to cell body loss, and possibly prior to the observation of frank cortical volume loss if validated in larger, longitudinal studies.

Our findings regarding age‐related macrostructural alterations in the eight selected cortical subregions are in good agreement with findings from prior imaging studies (Raz et al., 2005) demonstrating a greater degree of atrophy in the lateral prefrontal cortex, orbital frontal cortex, inferior parietal cortex, inferior temporal cortex, and hippocampus, with a comparatively smaller degree of volume loss in the fusiform cortex. The degree of volume loss within the entorhinal cortex was minimal (Knoops et al., 2012; Kurth et al., 2017; Raz et al., 2005), and the primary visual cortex remained stable in volume with advancing aging (Brewer & Barton, 2014; Crossland et al., 2008; Raz et al., 2005). Of note, our findings also showed that the entorhinal cortex exhibited no significant age‐related microstructural alterations as reflected in the SANDI metrics.

Considerable declines in intra‐soma signal fraction *f*_is_ and apparent soma radius *r*_s_ were observed across the cerebral cortex with advancing age. These alterations paralleled strong correlations with cortical volume across most of the major cortical lobes, indicating that reductions in cell body density and radius accompany age‐related tissue atrophy over the lifespan. Our results are in agreement with previous imaging studies showing frontal lobe‐predominant changes in cortical thickness and white matter microstructure in normal aging (Fan et al., 2019; Lebel et al., 2012; Madden et al., 2012; Raz et al., 2004; Salat et al., 2005). These studies highlighted the vulnerability of white matter within the frontal cortex to age‐related degeneration and used cortical atrophy as the primary measure of age‐related neurodegeneration. The occipital lobe was generally the region least affected by age‐related volume loss. In line with these findings, our results revealed a lower correlation between age and cortical volume in the occipital lobe, with substantial frontal‐predominant cortical volume loss in the aging brain. However, despite being the region with the lowest correlation with age, significant declines in intra‐soma signal fraction were observed within the occipital gray matter. Our findings suggest that the SANDI model may provide meaningful biomarkers to detect and characterize microstructural alterations within the aging brain, offering a promising pathway for the early identification and understanding of aging‐related effects on brain tissue microarchitecture.

The identification of spatial patterns of cortical microstructural alterations in normal aging and neurodegenerative disorders may help to uncover their underlying mechanisms. Notably, normal aging shows a distinct frontal predominant predilection, while Alzheimer's disease primarily affects the temporal lobe and limbic system (Madden et al., 2012; Minkova et al., 2017; Planche et al., 2022; Salat et al., 2010, 2011), as reflected by cortical thinning (Salat et al., 2011; Schmand et al., 2011), significant neuronal loss, and the presence of neurofibrillary tangles and amyloid plaques (Ossenkoppele et al., 2022; Thal et al., 2006), all linked to cognitive decline and symptoms (Marks et al., 2017; Scott et al., 2020). In our study of cognitively unimpaired adults, the correlations between intra‐soma signal fraction *f*_is_ and age in the inferior temporal cortex and hippocampus were less pronounced than in other cortical regions, and no significant correlation was observed between SANDI measures and age in the entorhinal cortex. Our results suggest that the SANDI measures applied to high‐gradient diffusion MRI data may not only be sensitive to the microstructural alterations in normal aging but also specific to the distribution of cellular changes in normal aging and potentially neurodegenerative diseases.

Two hypotheses may explain the increasing intra‐neurite signal fraction *f*_in_ with age in the major cortical lobes. First, the intra‐neurite signal fraction *f*_in_ reflects an authentic age‐related increase in anisotropic neurite‐like structures in gray matter, such as increased packing density or alterations in the orientation and integrity of neurites, with age leading to more anisotropic diffusion patterns, captured by the increasing intra‐neurite signal fraction *f*_in_. The increases in other independent measures of diffusion, FA from DTI and kurtoses from DKI, can support the interpretation of an increase in intra‐neurite signal fraction with age. Through additional quadratic regression analysis, the intra‐neurite signal fraction *f*_in_ followed an inverted U‐shaped curve, suggesting brain maturation followed by degeneration of the neurite compartment, with a peak age of 55 years. This trend might explain the overall increase in intra‐neurite signal fraction *f*_in_ with age. Second, the signal fractions of the three compartments in the SANDI model are the T_2_‐weighted MR signal fractions in each tissue compartment, not the absolute volume fraction values. The T_2_ relaxation times of the three compartments are expected to vary and change with age, potentially introducing biases in the derived SANDI metrics. Since the three signal fractions (*f*_in_, *f*_is_, and *f*_ec_) sum up to unity, one of them will inherently be linearly dependent on the other two. Therefore, the observed age‐related alteration of intra‐neurite signal fraction *f*_in_ in SANDI may not reflect the actual changes in absolute volume fraction of neurites (Dortch et al., 2013; Palombo et al., 2020). It may be attributed to the steep decrease in intra‐soma signal fraction *f*_is_ and increase in extracellular signal fraction *f*_ec_ with age. Their linear dependency necessitates careful interpretation of their age dependence. A previous study exploring SANDI metrics in healthy controls and multiple sclerosis patients reported a positive correlation between intra‐neurite signal fraction and age in the cortex using multiple regression analysis (Margoni et al., 2023). However, it contradicts the findings of a prior NODDI paper that indicated a widespread decrease in cortical neurite density (Gozdas et al., 2021). To estimate absolute volume fractions, The different T_2_ values in intra‐soma and intra‐neurite spaces could be considered in the model‐fitting (Gong et al., 2023) for future studies.

The increases in extracellular signal fraction *f*_ec_ and extracellular diffusivity *D*_ec_ seen with aging may reflect such factors as the breakdown of myelin (Bartzokis, 2004), astroglial dysfunction (Popov et al., 2021; Salas et al., 2020; Verkhratsky et al., 2021), and/or alterations in the microvasculature (Banks et al., 2021), contributing to increased extracellular space in the cortex and allowing water molecules to diffuse more freely in tissues.

The DTI and DKI metrics showed statistically significant correlations with age in the cortex. Traditionally, these models have been primarily used for the investigation of white matter integrity (Fieremans et al., 2011; Madden et al., 2012). In this study, FA showed a negative correlation with age in the overall white matter, consistent with prior studies that have attributed such findings to age‐related white matter degeneration and axonal disorganization (Beck et al., 2021; Westlye et al., 2010). Interestingly, FA showed a positive correlation with age in the cortex as a whole, especially in the frontal and parietal lobes, which is similar to findings in deep gray matter areas such as the caudate and putamen (Gong et al., 2014; Pfefferbaum et al., 2010). We observed an increase in MD with aging in the cortical gray matter, consistent with prior studies (Jeon et al., 2012; Salminen et al., 2016). In contrast, DTI metrics did not show a significant correlation with normalized cortical volume. All DKI metrics showed a positive correlation with age in the cortical gray matter. Although both DTI and DKI models demonstrated sensitivity to age‐related alterations in the major cortical regions, their lack of biological specificity makes their interpretation challenging. A major advantage of using a biophysical multi‐compartment model of diffusion such as SANDI is the ability to interpret the findings in the context of the underlying tissue constituents.

This study has several limitations. The SANDI model considers soma and neurites as impermeable and neglects water exchange between different compartments (Fieremans et al., 2010; Jelescu et al., 2022; Nilsson et al., 2010; Olesen et al., 2022), which may affect the accuracy of the SANDI metric estimation (Palombo et al., 2020). The effect of exchange on SANDI metric estimation has been simulated and shown to depend on diffusion time (Palombo et al., 2020). At short diffusion times ~10 ms, the estimated SANDI metrics are close to their expected values, whereas at long diffusion times ~80 ms, the estimated SANDI metrics deviate from expected due to exchange. To minimize the effect of exchange, prior studies have advised using the shortest accessible diffusion time, preferably less than 20 ms, in the diffusion MR acquisition for SANDI model fitting. A major benefit of using the 3T Connectome system for this study was the ability to achieve shorter diffusion times using strong gradients up to 300 mT/m. As high‐performance gradient systems become more widely and commercially available (Vachha & Huang, 2021), we anticipate that our results should be readily replicated on systems with comparable gradient strengths if sufficiently short diffusion times and high spatial resolution are used (Fan et al.^2017^; Foo et al., 2020; Huang et al., 2021). The significant associations between SANDI metrics and age were mainly identified using linear regression analysis in this study; however, the relationship between aging and gray matter microstructure is likely more complex as demonstrated by the quadratic regression analysis, and may not be fully characterized by a linear relationship as in the white matter (Beck et al., 2021; Burzynska et al., 2024; Kiely et al., 2022; Qian et al., 2020; Sullivan & Pfefferbaum, 2006; Westlye et al., 2010). The scope of this study was limited to imaging measures and age only; we did not explore the association between SANDI metrics and cognitive performance due to the lack of cognitive data in our cohort, although all older adults were screened for cognitive impairment. Studying the association between SANDI metrics and more comprehensive cognitive outcomes could provide valuable insights into the implications of the observed reduction in intra‐soma signal fraction and soma radius with cognitive function in normal aging and will be the topic of future studies on the latest high‐performance gradient systems (Huang et al., 2021). The stability and reliability of SANDI fitting can be influenced by the ranges assumed for the parameters. Future studies should further explore the impact of varying parameter ranges on SANDI metrics to ensure the robustness of the technique.

## 4 CONCLUSIONS

In this study, we showed age‐related alterations in gray matter microstructure in the cortex across the healthy adult lifespan using advanced biophysical modeling of diffusion MRI data acquired on the high‐gradient 3T Connectome MRI scanner. Lower intra‐soma signal fraction was significantly associated with older age throughout the major cortical lobes. Furthermore, of all the diffusion MRI measures studied here, the intra‐soma signal fraction showed the strongest and most significant association with normalized cortical volume. Regional cortical atrophy was accompanied by significant reductions in intra‐soma signal fraction and soma radius. Our findings suggest that high‐gradient diffusion MRI may have comparable or greater sensitivity to the underlying substrate of neurodegeneration in the aging brain than traditional macroscopic measures of neurodegeneration such as cortical volume and cortical thickness. The application of advanced biophysical modeling using high‐gradient diffusion MRI offers a powerful approach for noninvasively evaluating age‐related changes in gray matter microstructure across the lifespan and provides a valuable tool for studying neurological disorders, particularly as high‐performance gradient systems become commercially available in clinical MRI systems.

## 5 MATERIALS AND METHODS

### 5.1 Participants

A total of 72 cognitively unimpaired healthy subjects (19–85 years, 41.4 ± 17.9 years, 40 females) were recruited from the Massachusetts General Hospital and the local community. Here, we focused on evaluating age‐related changes in tissue microstructure in adults starting from the age of 19 years and onward, encompassing the transitional period from adolescence to adulthood, and extending throughout the entire adult lifespan. Participants with a history of neurological and psychiatric disorders were excluded from the study. All individuals provided informed consent prior to participation in the study, which was approved by the Institutional Review Board of Massachusetts General Brigham.

### 5.2 Data acquisition

The participants were scanned on the 3T Connectome MRI scanner (MAGNETOM CONNECTOM, Siemens Healthineers, Germany) equipped with a maximum gradient strength of 300 mT/m and a slew rate of 200 T/m/s. A customized 64‐channel phased array head coil was used for the signal reception (Keil et al., 2013). *T*_1_‐weighted anatomical images were acquired with 1 mm isotropic voxel size using a 3D multi‐echo magnetization‐prepared gradient echo (MEMPRAGE) sequence with the following parameters: repetition time (TR) = 2530 ms, echo time (TE) = 1.15, 3.03, 4.89, and 6.75 ms, inversion time (TI) = 1100 ms, generalized autocalibrating partially parallel acquisition (GRAPPA) acceleration factor = 3, and flip angle = 7°. DWI was acquired using a 2D diffusion‐weighted pulsed‐gradient spin‐echo echo‐planar imaging (DW‐PGSE‐EPI) sequence with a diffusion time (∆) = 19 ms and a gradient duration (𝛿) = 8 ms (Tian et al., 2022). Other DWI parameters were TR = 4000 ms, TE = 77 ms, 2 mm isotropic voxel size, partial Fourier = 6/8, GRAPPA acceleration factor = 2, simultaneous multislice (SMS) acceleration factor = 2, and anterior‐to‐posterior phase encoding direction. DWIs were acquired in 8 *b*‐values ranging from 0.05 to 6 ms/μm^2^ (0.05, 0.35, 0.8, 1.5, 2.4, 3.45, 4.75, and 6 ms/μm^2^). Interspersed *b* = 0 ms/μm^2^ images were acquired every 16 DWIs for signal normalization and motion correction. Thirty‐two diffusion encoding directions were acquired in a uniform distribution over the unit sphere for *b* < 2.3 ms/μm^2^, and 64 uniformly distributed diffusion encoding directions were acquired otherwise. Additionally, five *b* = 0 ms/μm^2^ images were acquired with reversed phase encoding direction (posterior‐to‐anterior) for susceptibility distortion correction.

### 5.3 Data processing

**FIGURE 6:**
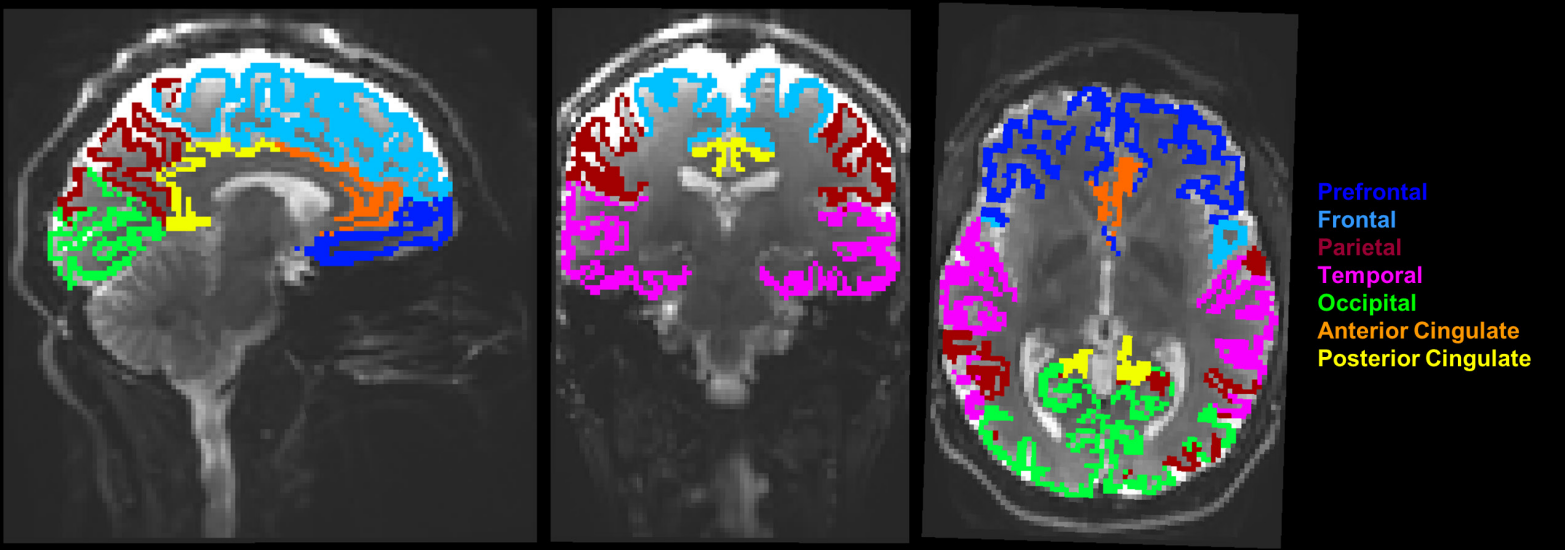
The major cortical lobar regions of interest (ROIs) from FreeSurfer registered to diffusion imaging space (non‐diffusion weighted image, *b* = 0 ms/μm^2^).

3D *T*_1_‐weighted MEMPRAGE data were processed with the use of FreeSurfer (Dale et al., 1999) (version 7.1.1; http://surfer.nmr.mgh.harvard.edu) for the segmentation of gray matter in the cerebral cortex (Figure 6) along with deep gray matter and white matter. The segmented cerebral cortex was then categorized into seven major cortical regions, including the prefrontal cortex, frontal lobe excluding the prefrontal cortex, parietal lobe, temporal lobe, occipital lobe, anterior cingulate gyrus, and posterior cingulate gyrus, based on the Destrieux atlas (Destrieux et al., 2010). Additionally, analyses in this study focused on eight specific cortical subregions that were affected in aging as examined in prior work (Raz et al., 2005), including the lateral prefrontal cortex (LPF), orbital frontal cortex (OF), inferior parietal cortex (IP), inferior temporal cortex (IT), fusiform cortex (FF), entorhinal cortex (EC), hippocampus (HC), and primary visual (pericalcarine) cortex (PV). The “mri_compute_volume_fractions” function in FreeSurfer was used to calculate the partial volume fractions for the cortex, subcortical gray matter, and white matter, with values of 0, 0.25, 0.5, 0.75, and 1. To minimize partial volume effects, partial volume‐weighted brain masks were created by combining the partial volume fraction map with the segmented brain regions. The estimation of volume was intended to convey the macrostructural characteristics of the corresponding region via the sum of values within the partial volume‐weighted mask. Volumes were normalized by dividing each volume by the total intracranial volume estimated by FreeSurfer.

An in‐house MATLAB (MathWorks, Natick, MA, USA) pipeline was used to preprocess the diffusion MRI data (Fan et al., 2016). All images were corrected for the gradient nonlinearity‐induced image warping and voxel shift (Jovicich et al., 2006). The “TOPUP” and “EDDY” functions in FSL (https://fsl.fmrib.ox.ac.uk) were used to correct the image distortion caused by susceptibility effect and eddy current as well as motion correction (Andersson et al., 2003; Andersson & Sotiropoulos, 2016). 3D *T*_1_‐weighted anatomical images were co‐registered to the averaged non‐DWI (*b* = 0 image) by using the “bbregister” function in FreeSurfer using a boundary‐based rigid body transformation of 6 degrees of freedom. The resulting transformation was applied to the partial volume‐weighted brain masks of the segmented cortical regions.

### 5.4 SANDI model analysis

In the SANDI model, the diffusion signal is modeled as receiving contributions from three compartments: the intra‐neurite space, intra‐soma space, and extracellular space (Palombo et al., 2020). The direction‐averaged signal $\tilde{S}\left(b\right)$ normalized by the non‐diffusion‐weighted *b* = 0 signal $S\left(0\right)$ is described as follows:

(1) $$\frac{\tilde{S}\left(b\right)}{S\left(0\right)}=f_{\text{in}}{\tilde{A}}_{\text{in}}\left(b,D_{\text{in}}\right)+f_{\text{is}}{\tilde{A}}_{\text{is}}\left(b,D_{\text{is}},r_{s}\right)+f_{\mathrm{ec}}{\tilde{A}}_{\mathrm{ec}}\left(b,D_{\mathrm{ec}}\right),$$

where intra‐neurite signal fraction *f*_in_, intra‐soma signal fraction *f*_is_, and extracellular signal fraction *f*_ec_ (*f*_in_ + *f*_is_ + *f*_ec_ = 1) are the T_2_‐weighted signal fractions in the intra‐neurite, intra‐soma, and extracellular space, respectively. ${\tilde{A}}_{\text{in}}$ represents the normalized direction‐averaged signal within the neurites, which are modeled as impermeable sticks with a parallel diffusivity *D*_in_ and negligible perpendicular diffusivity (Palombo et al., 2020). ${\tilde{A}}_{\text{is}}$ represents the normalized direction‐averaged signal within the soma, which are modeled as impermeable spheres with an apparent soma radius *r*_s_ and intrinsic diffusivity *D*_is_ (Palombo et al., 2020). ${\tilde{A}}_{\mathrm{ec}}$ represents the normalized direction‐averaged signal in the extracellular space, which is modeled as an isotropic Gaussian diffusion compartment with an apparent diffusivity *D*_ec_ (Palombo et al., 2020).

The SANDI metrics were calculated by fitting Equation (1) to the directionally averaged (spherical mean) signals of the magnitude data from the multi‐shell DWIs using the SANDI MATLAB toolbox (https://github.com/palombom/SANDI‐Matlab‐Toolbox). The SANDI toolbox employs a random forest regression algorithm (*TreeBagger* in MATLAB) with 200 trees. To train the random forest regressor, intra‐soma diffusivity *D*_is_ was fixed at 3 μm^2^/ms, and the five unknown parameters were uniformly distributed within the specific ranges as follows: extracellular diffusivity *D*_ec_ = [0.25, 3] μm^2^/ms, intra‐neurite diffusivity *D*_in_ = [0.25, 3] μm^2^/ms, soma radius *r*_s_ = [1, 12] μm, intra‐neurite signal fraction *f*_in_ = [0, 1], and intra‐soma signal fraction *f*_is_ = [0, 1] (Palombo et al., 2020). The training data consisted of normalized simulated signals using 10^5^ various combinations of the parameters. The signal‐to‐noise ratio (SNR) of *b* = 0 images was estimated from the noise map generated by Marchenko–Pastur principal component analysis (MP‐PCA) denoising using the “dwidenoise” function in MRtrix (https://www.mrtrix.org) (Veraart et al., 2016). The normalized noise level, defined as the inverse of SNR of *b* = 0 images, was applied to the simulated signals based on Rician noise statistics. The five independent tissue parameters [*f*_is_, *f*_in_, *r*_s_, *D*_ec_, and *D*_in_] were fitted in each voxel to generate SANDI metric maps. The extracellular signal fraction *f*_ec_ was calculated based on the constraint, *f*_is_ + *f*_in_ + *f*_ec_ = 1, different from that in the referenced paper (Palombo et al., 2020), where *f*_is_ + *f*_in_ = 1. These fractions are apparent, as they are significantly affected by the TE due to the differential T_2_ relaxation times of the tissue compartments. Additionally, the T_2_ relaxation times of the three compartments are expected to vary and change with age, potentially introducing biases in the derived SANDI metrics. The age‐related changes in one fraction must be considered in the context of the changes in the other two fractions.

The representative SANDI metrics maps were averaged over all subjects for visualization using the “population_template” function in MRtrix.

### 5.5 DTI and DKI analysis

DTI and DKI metrics (Jensen et al., 2005) were calculated in the cerebral white matter and major cortical regions using a subset of the overall diffusion data. For the DTI analysis, the data acquired with *b* = 0 and 0.8 ms/μm^2^ were used to calculate DTI metrics [FA, AD, RD, and MD], employing the “dwi2tensor” and “tensor2metric” functions in MRtrix. Similarly, for the DKI analysis, the data acquired with *b* = 0, 0.8, 1.5, 2.4, and 3.45 ms/μm^2^ were used to calculate DKI metrics [AK, RK, and MK] using the same functions in MRtrix.

### 5.6 Noise propagation of SANDI model

To assess the model fit of SANDI to the high‐gradient diffusion MRI data, the normalized diffusion signals $S\left(b\right)$ were generated over 1000 iterations using the forward model (1) of SANDI with randomly distributed parameters as follows: *D*_ec_ = [0.5, 1.5] μm^2^/ms, *D*_in_ = [1, 2] μm^2^/ms, *r*_s_ = [5, 11] μm, *f*_in_ = [0.1, 0.5], and *f*_is_ = [0.1, 0.5]. Rician noise was applied to the signals $S\left(b\right)$ at two different SNR levels (10^10^ and 50) (Afzali et al., 2021). The SNR levels were chosen to approximate nearly noise‐free signals (SNR = 10^10^) and the in vivo DWIs (SNR ≈ 50):

$$S_{n}=\sqrt{{\left(S+N_{r}\left(0,\sigma \right)\right)}^{2}+{\left(N_{i}\left(0,\sigma \right)\right)}^{2}},$$

where $S_{n}$ is the normalized diffusion signal with Rician noise. $N_{r}$ and $N_{i}$ are normal‐distributed noise in real and imaginary parts with a normalized noise level σ≡ 1/SNR. The SANDI model was fitted to normalized diffusion signals using the SANDI MATLAB toolbox, with the same parameter range settings as the in vivo diffusion MRI protocol. The accuracy of the SANDI fitting by random forest regression as assessed through the noise propagation experiments is shown in the Supporting Information.

### 5.7 Statistical analysis

Statistical analysis was conducted using MATLAB. Kolmogorov–Smirnov test was used for the normality test of the data. The correlation between the age and normalized volume of each cortical region, deep gray matter, and cerebral white matter was assessed using Pearson's correlation coefficient analysis. The average values of SANDI, DTI, and DKI metrics were calculated in each selected ROI, in which only voxels with partial volume weighting of cerebral gray matter, deep gray matter, and cerebral white matter greater than 0.5 were included to ensure a focus on regions with predominant content of each type of matter. The average values of SANDI metrics in the major cortical regions were compared among the three age groups (a young adult group, a middle‐aged group, and an older adult group) using analysis of covariance (ANCOVA) with sex as a covariate. A post hoc analysis involving an unpaired t‐test was performed to compare the three groups. Pearson's correlation coefficients were used to evaluate the correlations between diffusion metrics [SANDI, DTI and DKI] and age, and the correlations between SANDI metrics and the normalized volume in each major cortical region and cortical subregion. Pearson's correlations between SANDI metrics and age and between SANDI metrics and normalized volume were also investigated in the deep gray matter and cerebral white matter. Pearson's correlation coefficients and the corresponding *p*‐values for correlations between SANDI metrics and age and between SANDI metrics and normalized cortical volume for each gyrus of the brain were projected onto a common template of the inflated cortical surface using the FreeSurfer functions “mri_vol2surf” and “mri_surf2surf” for visualization purposes. All Pearson's correlation coefficients were analyzed after controlling for sex as part of a partial correlation analysis (Salat et al., 1997). To study the influence of free water signal on the correlation analysis of SANDI metrics with age and normalized volume, Pearson's correlations between free water signal fraction derived from NODDI and age were examined, along with the correlations of SANDI metrics with age and normalized volume, incorporating free water signal fraction as an additional covariate. Quadratic regression was conducted to explore the nonlinear trends of SANDI, DTI, and DKI metrics with aging in cerebral cortex and cerebral white matter. *R* ^2^ values were measured to assess the goodness of fit of linear and quadratic regressions. We adjusted the *p*‐values for multiple comparisons using the false discovery rate (reported as FDR‐*p*). The difference between the two correlation coefficients was determined by calculating *z*‐scores through Fisher's *r*‐to‐*z* transformation. The threshold for statistical significance was set at *α* = 0.05.

## AUTHOR CONTRIBUTIONS

*Study design*: HHL, DHS, ECK, and SYH. *Study conduct and data collection*: HL, LE, KG, AWR, and SYH. *Data analysis*: HL, HHL, YM, LE, QT, EAK, and SYH. *Data interpretation*: HL, HHL, QT, EAK, DHS, ECK, SYH. *Drafting the manuscript*: HL, HHL, and SYH.

## CONFLICT OF INTEREST STATEMENT

The author(s) declared no potential conflicts of interest with respect to the research, authorship, and/or publication of this article.

## Supporting information


Data S1.

## Data Availability

The data and code used in the study are available upon direct request as well as the conditions for its sharing or re‐use.
