# Supplementary material for: Age‐related alterations in human cortical microstructure across the lifespan: Insights from high‐gradient diffusion MRI
Source: Aging Cell. 2024 Aug 8;23(11):e14267. doi: 10.1111/acel.14267 (PMC11561659; doi:10.1111/acel.14267)
Supplement: Supplementary file 1 — Data S1. [file ACEL-23-e14267-s001.docx]

**Age-related alterations in human cortical microstructure across the lifespan: Insights from high-gradient diffusion MRI**

Hansol Lee^1,†^, Hong-Hsi Lee^1,†^, Yixin Ma^1^, Laleh Eskandarian^1^, Kyla Gaudet^1^, Qiyuan Tian^1^, Eva A. Krijnen^2,3^, Andrew W. Russo^2^, Leah G. Capuano^2^, David H. Salat^1^, Eric C. Klawiter^2^, and Susie Y. Huang^1,4*^

^1^Department of Radiology, Athinoula A. Martinos Center for Biomedical Imaging, Massachusetts General Hospital, Charlestown, Massachusetts, USA

^2^Department of Neurology, Massachusetts General Hospital, Harvard Medical School, Boston, Massachusetts, USA

^3^MS Center Amsterdam, Anatomy and Neurosciences, Amsterdam Neuroscience, Amsterdam UMC location VUmc, Amsterdam, The Netherlands

^4^Harvard-MIT Division of Health Sciences and Technology, Massachusetts Institute of Technology, Cambridge, Massachusetts, USA

† These authors contributed equally to this work.

* Corresponding authors:

Susie Y. Huang

Address: Department of Radiology, Athinoula A. Martinos Center for Biomedical Imaging, Massachusetts General Hospital, 149 13th Street, Room 2301, Charlestown, Massachusetts 02129, USA

E-mail : [susie.huang@mgh.harvard.edu](mailto:susie.huang@mgh.harvard.edu)

***Supplementary Results***

Here, we showed the accuracy of the SANDI fitting (random forest regression) through noise propagation (**Supplementary Figure 8**). In this simulation, we tested the SANDI fitting using the almost noise-free signals with an SNR of 10^10^ and noisy signals with an SNR of 50. For the almost noise-free signals, the fitted extracellular diffusivity *D_ec_* was underestimated, and the fitted intra-neurite diffusivity *D_in_* was not just underestimated but also remained constant for all ground truth values. The fitted soma radius *r_s_* was positively correlated with the ground truth values, albeit with some biases. The fitted extracellular signal fraction *f_ec_* was overestimated for ground truth values < 0.6. The fitted intra-neurite signal fraction *f_in_* was mostly underestimated with some biases, and the fitted intra-soma signal fraction *f_is_* almost remained constant at around 0.22. For the noisy signals with an SNR of 50, the fitted extracellular diffusivity *D_ec_* and intra-neurite signal fraction *f_in_* coincided with the ground truth values. The fitted soma radius *r_s_* and extracellular signal fraction *f_ec_* were positively correlated with ground truth values, though with some biases. In contrast, the fitted intra-neurite diffusivity *D_in_* and intra-soma signal fraction *f_is_* remained constant for all ground truth values.


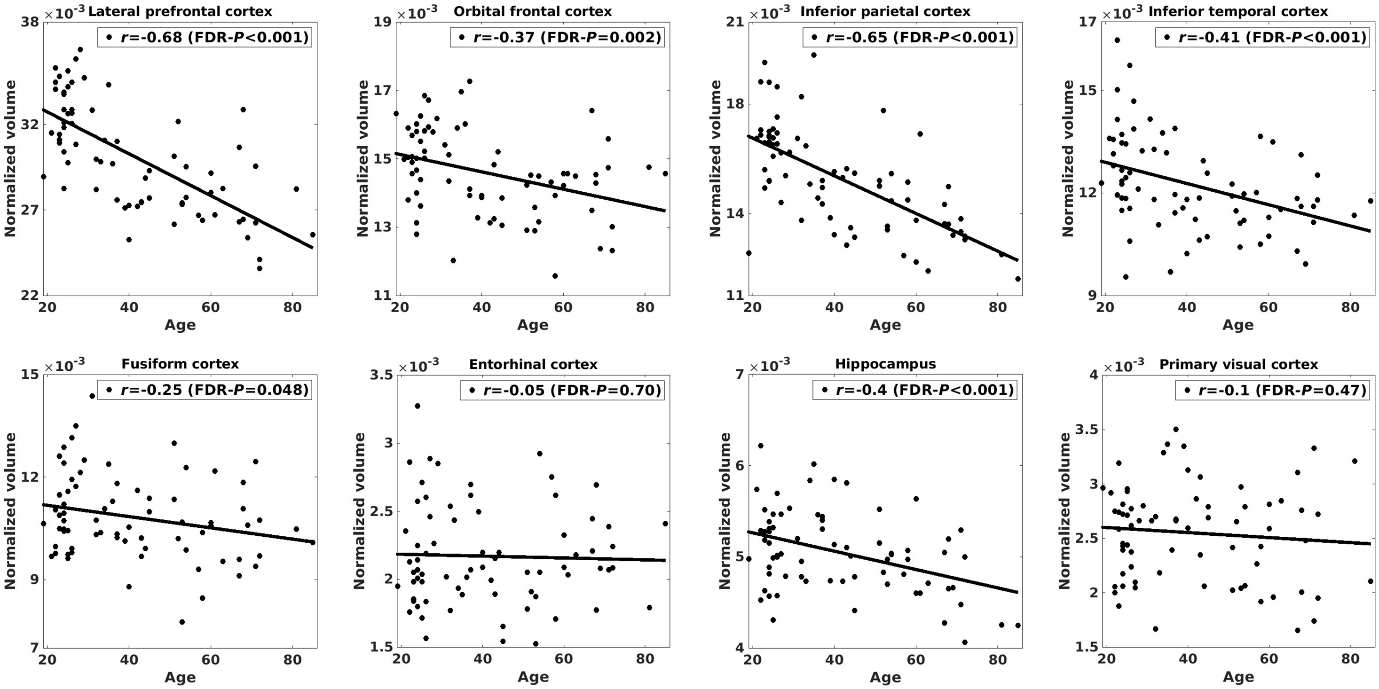


**Supplementary Figure 1. Correlations between age and normalized cortical volume in eight selected cortical subregions.** *r* value is Pearson’s correlation coefficient with *P*-value after correcting for multiple comparisons using the FDR.

**Supplementary Table 1. Comparisons of SANDI metrics in the entire cerebral cortex across different age groups**

|  | **All**  **Participants**  **N = 72** | **Young**  **(under 34)**  **N = 33** | **Middle**  **(35 to 54)**  **N = 21** | **Older**  **(over 55)**  **N = 18** | **ANCOVA** | | **FDR-*P* value** | | |
| --- | --- | --- | --- | --- | --- | --- | --- | --- | --- |
|  |  |  |  |  | **F value** | **P value** | **Young *vs.* Middle** | **Young *vs.* Old** | **Middle *vs.* Old** |
| *f_is_* | 0.42 $\pm$ 0.02 | 0.43 $\pm$ 0.01 | 0.41 $\pm$ 0.02 | 0.40 $\pm$ 0.01 | 36.5 | <0.001* | **<0.001*** | **<0.001*** | **<0.001*** |
| *f_in_* | 0.17 $\pm$ 0.01 | 0.16 $\pm$ 0.01 | 0.17 $\pm$ 0.01 | 0.17 $\pm$ 0.01 | 8.5 | <0.001* | **0.003*** | **0.002*** | 0.92 |
| *f_ec_* | 0.41 $\pm$ 0.02 | 0.40 $\pm$ 0.02 | 0.41 $\pm$ 0.02 | 0.43 $\pm$ 0.02 | 14.9 | <0.001* | **0.01*** | **<0.001*** | **0.02*** |
| *r_s_* ($\mu$m) | 9.26 $\pm$ 0.14 | 9.30 $\pm$ 0.16 | 9.28 $\pm$ 0.08 | 9.17 $\pm$ 0.11 | 6.2 | 0.003* | 0.57 | **0.003*** | **0.02*** |
| *D_ec_* ($\mu$m^2^/ms) | 1.60 $\pm$ 0.09 | 1.57 $\pm$ 0.09 | 1.59 $\pm$ 0.07 | 1.66 $\pm$ 0.07 | 6.2 | 0.003* | 0.42 | **0.003*** | **0.02*** |
| *D_in_* ($\mu$m^2^/ms) | 1.83 $\pm$ 0.02 | 1.83 $\pm$ 0.02 | 1.83 $\pm$ 0.02 | 1.82 $\pm$ 0.02 | 2.1 | 0.14 | 0.97 | 0.14 | 0.14 |

Data are shown as mean $\pm$ standard deviation.

FDR-*P* = *P*-values from unpaired t-test after correcting for multiple comparisons using the FDR.

*: *P*-values < 0.05.

**Supplementary Table 2. SANDI metrics in the major cortical regions**

|  | **Cerebral WM** | **Entire cerebral**  **cortex** | **Prefrontal** | **Frontal** | **Parietal** | **Temporal** | **Occipital** | **Anterior**  **Cingulate** | **Posterior**  **Cingulate** |
| --- | --- | --- | --- | --- | --- | --- | --- | --- | --- |
| *f_is_* | 0.27 $\pm$ 0.02 | 0.42 $\pm$ 0.02 | 0.42 $\pm$ 0.02 | 0.43 $\pm$ 0.02 | 0.43 $\pm$ 0.02 | 0.41 $\pm$ 0.02 | 0.40 $\pm$ 0.03 | 0.45 $\pm$ 0.02 | 0.40 $\pm$ 0.03 |
| *f_in_* | 0.42 $\pm$ 0.02 | 0.17 $\pm$ 0.01 | 0.16 $\pm$ 0.01 | 0.17 $\pm$ 0.01 | 0.16 $\pm$ 0.01 | 0.18 $\pm$ 0.01 | 0.18 $\pm$ 0.01 | 0.15 $\pm$ 0.01 | 0.21 $\pm$ 0.02 |
| *f_ec_* | 0.32 $\pm$ 0.02 | 0.41 $\pm$ 0.02 | 0.42 $\pm$ 0.02 | 0.40 $\pm$ 0.02 | 0.41 $\pm$ 0.03 | 0.41 $\pm$ 0.02 | 0.42 $\pm$ 0.02 | 0.40 $\pm$ 0.01 | 0.39 $\pm$ 0.03 |
| *r_s_* ($\mu$m) | 8.88 $\pm$ 0.10 | 9.26 $\pm$ 0.15 | 9.23 $\pm$ 0.15 | 9.14 $\pm$ 0.19 | 9.32 $\pm$ 0.18 | 9.29 $\pm$ 0.13 | 9.31 $\pm$ 0.11 | 9.36 $\pm$ 0.16 | 9.21 $\pm$ 0.17 |
| *D_ec_* ($\mu$m^2^/ms) | 1.59 $\pm$ 0.09 | 1.60 $\pm$ 0.07 | 1.59 $\pm$ 0.10 | 1.50 $\pm$ 0.13 | 1.61 $\pm$ 0.12 | 1.63 $\pm$ 0.09 | 1.73 $\pm$ 0.11 | 1.39 $\pm$ 0.11 | 1.63 $\pm$ 0.15 |
| *D_in_* ($\mu$m^2^/ms) | 1.95 $\pm$ 0.03 | 1.83 $\pm$ 0.03 | 1.80 $\pm$ 0.02 | 1.81 $\pm$ 0.03 | 1.83 $\pm$ 0.03 | 1.84 $\pm$ 0.02 | 1.86 $\pm$ 0.02 | 1.79 $\pm$ 0.04 | 1.87 $\pm$ 0.03 |

Data are shown as mean $\pm$ standard deviation.

**Supplementary Table 3. Correlations between age and SANDI metrics in the eight cortical subregions**

|  | **LPF** | **OF** | **IP** | **IT** | **FF** | **EC** | **HC** | **PV** |
| --- | --- | --- | --- | --- | --- | --- | --- | --- |
| *f_is_* | **-0.60**  **(<0.001*)**  **(<0.001*)** | **-0.57**  **(<0.001*)**  **(<0.001*)** | **-0.65**  **(<0.001*)**  **(<0.001*)** | **-0.35**  **(0.003*)**  **(0.007*)** | **-0.57**  **(<0.001*)**  **(<0.001*)** | -0.15  (0.20)  (0.27) | **-0.46**  **(<0.001*)**  **(<0.001*)** | **-0.64**  **(<0.001*)**  **(<0.001*)** |
| *f_in_* | 0.18  (0.12)  (0.17) | 0.14  (0.25)  (0.31) | **0.28**  **(0.02*)**  **(0.03*)** | 0.03  (0.80)  (0.82) | 0.10  (0.42)  (0.47) | 0.13  (0.30)  (0.37) | **-0.26**  **(0.03*)**  **(0.04*)** | -0.11  (0.34)  (0.41) |
| *f_ec_* | **0.51**  **(<0.001*)**  **(<0.001*)** | **0.60**  **(<0.001*)**  **(<0.001*)** | **0.48**  **(<0.001*)**  **(<0.001*)** | **0.39**  **(<0.001*)**  **(0.002*)** | **0.54**  **(<0.001*)**  **(<0.001*)** | 0.08  (0.49)  (0.54) | **0.58**  **(<0.001*)**  **(<0.001*)** | **0.57**  **(<0.001*)**  **(<0.001*)** |
| *r_s_* ($\mu$m) | **-0.45**  **(<0.001*)**  **(<0.001*)** | **-0.31**  **(0.008*)**  **(0.01*)** | **-0.33**  **(0.006*)**  **(0.01*)** | -0.11  (0.37)  (0.42) | **-0.39**  **(<0.001*)**  **(0.002*)** | 0.11  (0.36)  (0.42) | **-0.49**  **(<0.001*)**  **(<0.001*)** | **-0.49**  **(<0.001*)**  **(<0.001*)** |
| *D_ec_* ($\mu$m^2^/ms) | **0.34**  **(0.004*)**  **(0.008*)** | **0.30**  **(0.01*)**  **(0.02*)** | **0.34**  **(0.004*)**  **(0.008*)** | **0.38**  **(0.001*)**  **(0.003*)** | **0.34**  **(0.004*)**  **(0.007*)** | -0.03  (0.78)  (0.81) | **0.32**  **(0.006*)**  **(0.01*)** | **0.31**  **(0.009*)**  **(0.01*)** |
| *D_in_* ($\mu$m^2^/ms) | **-0.33**  **(0.005*)**  **(0.009*)** | -0.08  (0.53)  (0.57) | -0.08  (0.52)  (0.57) | 0.07  (0.57)  (0.60) | **-0.27**  **(0.02*)**  **(0.03*)** | -0.004  (0.97)  (0.97) | **-0.42**  **(<0.001*)**  **(<0.001*)** | **-0.42**  **(<0.001*)**  **(<0.001*)** |

Data are reported as Pearson’s correlation coefficient with corresponding uncorrected *P*-values (first pair of parentheses) and FDR-corrected *P*-values (second pair of parentheses). * with the bold values denotes statistical significance (*P* < 0.05 or FDR-*P* < 0.05) in Pearson’s correlation coefficient analysis. LPF = Lateral prefrontal cortex; OF = Orbital frontal cortex; IP = Inferior parietal cortex; IT = Inferior temporal cortex; FF = Fusiform cortex; EC = Entorhinal cortex; HC = Hippocampus; PV = Primary visual (pericalcarine) cortex.

**Supplementary Table 4. Correlations between SANDI metrics and normalized cortical volume of each region in the eight cortical subregions**

|  | **LPF** | **OF** | **IP** | **IT** | **FF** | **EC** | **HC** | **PV** |
| --- | --- | --- | --- | --- | --- | --- | --- | --- |
| *f_is_* | **0.62**  **(<0.001*)**  **(<0.001*)** | 0.18  (0.12)  (0.27) | **0.52**  **(<0.001*)**  **(<0.001*)** | 0.24  **(0.04*)**  (0.15) | 0.11  (0.35)  (0.51) | -0.17  (0.16)  (0.32) | 0.09  (0.46)  (0.59) | 0.01  (0.92)  (0.96) |
| *f_in_* | -0.21  (0.09)  (0.24) | -0.01  (0.94)  (0.96) | -0.16  (0.19)  (0.35) | -0.18  (0.13)  (0.27) | -0.18  (0.13)  (0.27) | 0.10  (0.42)  (0.56) | 0.20  (0.10)  (0.25) | 0.12  (0.32)  (0.49) |
| *f_ec_* | **-0.52**  **(<0.001*)**  **(<0.001*)** | -0.23  (0.06)  (0.20) | **-0.42**  **(<0.001*)**  **(0.004*)** | -0.13  (0.26)  (0.44) | -0.01  (0.94)  (0.96) | 0.12  (0.30)  (0.48) | -0.20  (0.09)  (0.25) | -0.05  (0.67)  (0.82) |
| *r_s_* ($\mu$m) | **0.41**  **(<0.001*)**  **(0.004*)** | 0.14  (0.25)  (0.44) | 0.19  (0.11)  (0.27) | 0.19  (0.12)  (0.27) | 0.02  (0.87)  (0.96) | -0.20  (0.10)  (0.25) | 0.17  (0.17)  (0.32) | 0.04  (0.75)  (0.88) |
| *D_ec_* ($\mu$m^2^/ms) | -0.26  **(0.03*)**  (0.12) | -0.01  (0.95)  (0.96) | -0.21  (0.07)  (0.23) | -0.12  (0.34)  (0.51) | 0.09  (0.45)  (0.59) | 0.26  **(0.03*)**  (0.12) | -0.02  (0.90)  (0.96) | -0.21  (0.08)  (0.24) |
| *D_in_* ($\mu$m^2^/ms) | **0.37**  **(0.001*)**  **(0.01*)** | 0.12  (0.30)  (0.48) | 0.14  (0.26)  (0.44) | -0.02  (0.89)  (0.96) | -0.11  (0.36)  (0.51) | -0.10  (0.40)  (0.54) | 0.28  **(0.02*)**  (0.10) | -0.02  (0.84)  (0.96) |

Data are reported as Pearson’s correlation coefficient with corresponding uncorrected *P*-values (first pair of parentheses) and FDR-corrected *P*-values (second pair of parentheses). * with the bold values denotes statistical significance (*P* < 0.05 or FDR-*P* < 0.05) in Pearson’s correlation coefficient analysis. LPF = Lateral prefrontal cortex; OF = Orbital frontal cortex; IP = Inferior parietal cortex; IT = Inferior temporal cortex; FF = Fusiform cortex; PV = Primary visual (pericalcarine) cortex; HC = Hippocampus; EC = Entorhinal cortex.


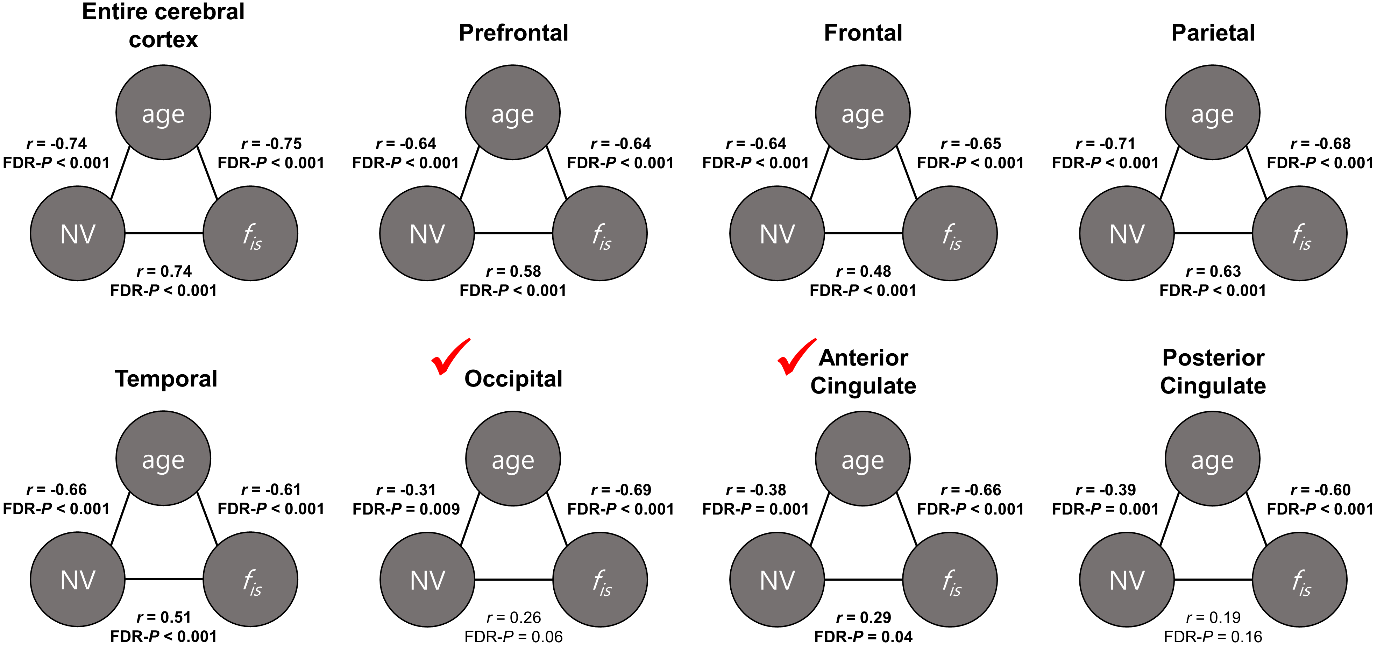


**Supplementary Figure 2. Correlations between the age, normalized volume, and *f_is_* of the SANDI metrics within the major cortical regions.** The red check mark indicates a significant difference in the correlation between age and *f_is_* compared to the correlation between age and normalized volume. NV = normalized volume.


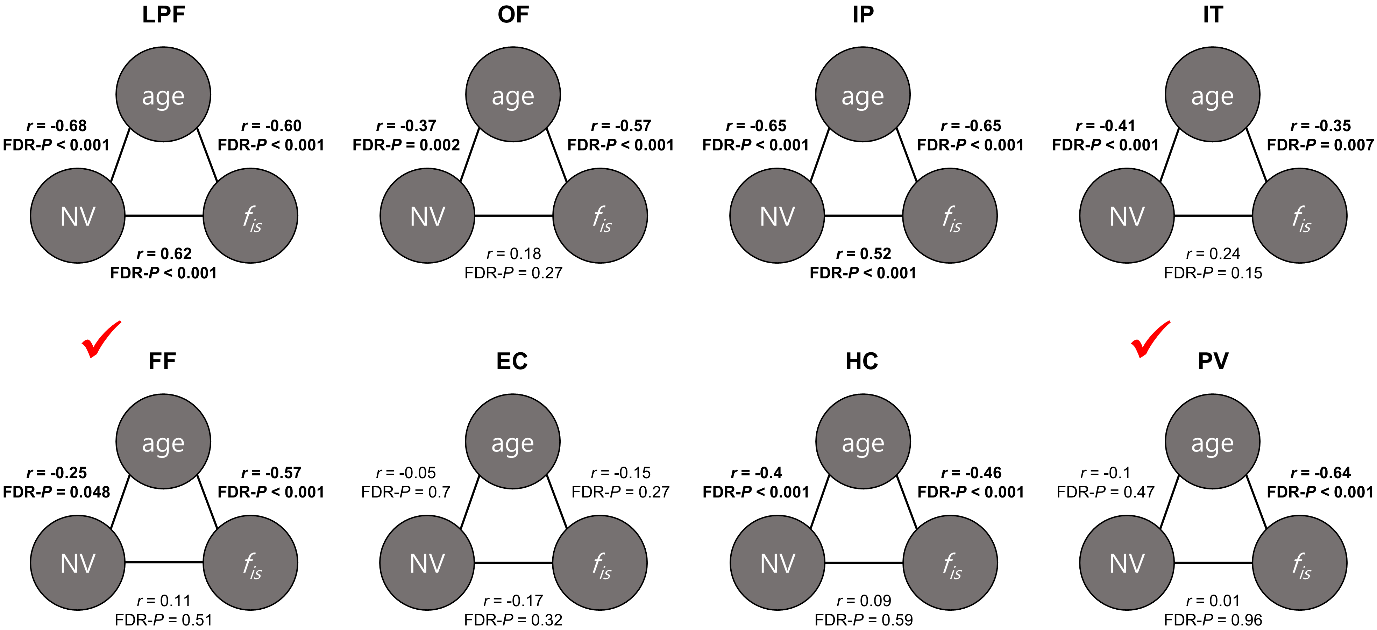


**Supplementary Figure 3. Correlations between the age, normalized volume, and *f_is_* of the SANDI metrics within the eight cortical subregions.** The red check mark indicates a significant difference in the correlation between age and *f_is_* compared to the correlation between age and normalized volume. NV = normalized volume; LPF = Lateral prefrontal cortex; OF = Orbital frontal cortex; IP = Inferior parietal cortex; IT = Inferior temporal cortex; FF = Fusiform cortex; PV = Primary visual (pericalcarine) cortex; HC = Hippocampus; EC = Entorhinal cortex.

**Supplementary Table 5. Correlations between *f_iso_* derived from NODDI and age and between *f_iso_* derived from NODDI and normalized volume**

|  | **Cerebral WM** | **Entire cerebral**  **cortex** | **Prefrontal** | **Frontal** | **Parietal** | **Temporal** | **Occipital** | **Anterior**  **Cingulate** | **Posterior**  **Cingulate** |
| --- | --- | --- | --- | --- | --- | --- | --- | --- | --- |
| *Age* | **0.25**  **(0.04*)**  **(0.047*)** | **0.65**  **(<0.001*)**  **(<0.001*)** | **0.52**  **(<0.001*)**  **(<0.001*)** | **0.56**  **(<0.001*)**  **(<0.001*)** | **0.57**  **(<0.001*)**  **(<0.001*)** | **0.51**  **(<0.001*)**  **(<0.001*)** | **0.62**  **(<0.001*)**  **(<0.001*)** | **0.61**  **(<0.001*)**  **(<0.001*)** | **0.48**  **(<0.001*)**  **(<0.001*)** |
| *Normalized volume* | **-0.31**  **(0.008*)**  **(0.01*)** | **-0.61**  **(<0.001*)**  **(<0.001*)** | **-0.38**  **(<0.001*)**  **(0.001*)** | **-0.36**  **(0.002*)**  **(0.003*)** | **-0.51**  **(<0.001*)**  **(<0.001*)** | **-0.40**  **(<0.001*)**  **(0.001*)** | -0.24  **(0.046*)**  (0.05) | -0.08  (0.51)  (<0.001*) | -0.15  (0.22)  (<0.001*) |

Data are reported as Pearson’s correlation coefficient with corresponding uncorrected *P*-values (first pair of parentheses) and FDR-corrected *P*-values (second pair of parentheses). * with the bold values denotes statistical significance (*P* < 0.05 or FDR-*P* < 0.05) in Pearson’s correlation coefficient analysis. NODDI = neurite orientation dispersion and density imaging.

**Supplementary Table 6. Correlations between age and SANDI metrics with *f_iso_* derived from NODDI as an additional covariate**

|  | **Cerebral WM** | **Entire cerebral**  **cortex** | **Prefrontal** | **Frontal** | **Parietal** | **Temporal** | **Occipital** | **Anterior**  **Cingulate** | **Posterior**  **Cingulate** |
| --- | --- | --- | --- | --- | --- | --- | --- | --- | --- |
| *f_is_* | -0.01  (0.92)  (0.92) | **-0.50**  **(<0.001*)**  **(<0.001*)** | **-0.45**  **(<0.001*)**  **(<0.001*)** | **-0.43**  **(<0.001*)**  **(0.001*)** | **-0.45**  **(<0.001*)**  **(<0.001*)** | **-0.41**  **(<0.001*)**  **(0.002*)** | **-0.41**  **(<0.001*)**  **(0.002*)** | **-0.38**  **(0.001*)**  **(0.006*)** | **-0.42**  **(<0.001*)**  **(0.002*)** |
| *f_in_* | -0.17  (0.15)  (0.28) | **0.48**  **(<0.001*)**  **(<0.001*)** | 0.25  **(0.04*)**  (0.09) | **0.48**  **(<0.001*)**  **(<0.001*)** | **0.36**  **(0.002*)**  **(0.01*)** | **0.28**  **(0.02*)**  **(0.049*)** | **0.34**  **(0.004*)**  **(0.02*)** | **0.30**  **(0.01*)**  **(0.04*)** | 0.23  (0.05)  (0.12) |
| *f_ec_* | 0.23  (0.05)  (0.11) | 0.16  (0.18)  (0.29) | **0.34**  **(0.004*)**  **(0.01*)** | 0.01  (0.90)  (0.92) | 0.15  (0.21)  (0.31) | 0.22  (0.06)  (0.13) | 0.17  (0.16)  (0.29) | 0.11  (0.36)  (0.45) | **0.34**  **(0.004*)**  **(0.02*)** |
| *r_s_* ($\mu$m) | -0.15  (0.20)  (0.30) | **-0.29**  **(0.01*)**  **(0.04*)** | **-0.30**  **(0.01*)**  **(0.04*)** | **-0.48**  **(<0.001*)**  **(<0.001*)** | **-0.32**  **(0.006*)**  **(0.02*)** | -0.17  (0.15)  (0.28) | -0.16  (0.19)  (0.29) | **-0.45**  **(<0.001*)**  **(<0.001*)** | -0.27  **(0.03*)**  (0.06) |
| *D_ec_* ($\mu$m^2^/ms) | 0.12  (0.33)  (0.43) | 0.10  (0.43)  (0.50) | 0.20  (0.10)  (0.20) | 0.11  (0.37)  (0.46) | 0.01  (0.92)  (0.92) | 0.12  (0.31)  (0.42) | 0.02  (0.18)  (0.29) | 0.10  (0.42)  (0.50) | 0.06  (0.60)  (0.66) |
| *D_in_* ($\mu$m^2^/ms) | -0.11  (0.35)  (0.45) | -0.04  (0.73)  (0.79) | -0.08  (0.49)  (0.55) | -0.16  (0.18)  (0.29) | -0.17  (0.15)  (0.28) | 0.09  (0.48)  (0.55) | -0.16  (0.86)  (0.91) | -0.12  (0.31)  (0.43) | -0.15  (0.21)  (0.30) |

Data are reported as Pearson’s correlation coefficient with corresponding uncorrected *P*-values (first pair of parentheses) and FDR-corrected *P*-values (second pair of parentheses). * with the bold values denotes statistical significance (*P* < 0.05 or FDR-*P* < 0.05) in Pearson’s correlation coefficient analysis. NODDI = Neurite orientation dispersion and density imaging.

**Supplementary Table 7. Correlations between SANDI metrics and normalized volume with *f_iso_* derived from NODDI as an additional covariate**

|  | **Cerebral WM** | **Entire cerebral**  **cortex** | **Prefrontal** | **Frontal** | **Parietal** | **Temporal** | **Occipital** | **Anterior**  **Cingulate** | **Posterior**  **Cingulate** |
| --- | --- | --- | --- | --- | --- | --- | --- | --- | --- |
| *f_is_* | **-0.31**  **(0.008*)**  **(0.03*)** | **0.53**  **(<0.001*)**  **(<0.001*)** | **0.47**  **(<0.001*)**  **(<0.001*)** | **0.33**  **(0.005*)**  **(0.02*)** | **0.43**  **(<0.001*)**  **(0.002*)** | **0.35**  **(0.003*)**  **(0.01*)** | 0.11  (0.35)  (0.57) | **0.35**  **(0.003*)**  **(0.01*)** | 0.12  (0.34) (0.57) |
| *f_in_* | **0.41**  **(<0.001*)**  **(0.004*)** | **-0.49**  **(<0.001*)**  **(<0.001*)** | -0.23  (0.06)  (0.13) | **-0.29**  **(0.02*)**  **(0.0497*)** | -0.28  **(0.02*)**  (0.05) | **-0.29**  **(0.01*)**  **(0.049*)** | -0.06  (0.62)  (0.71) | **-0.31**  **(0.008*)**  **(0.03*)** | -0.10  (0.42)  (0.61) |
| *f_ec_* | **-0.29**  **(0.02*)**  **(0.497*)** | -0.19  (0.11)  (0.25) | **-0.38**  **(0.001*)**  **(0.007*)** | -0.10  (0.41)  (0.61) | -0.19  (0.11)  (0.25) | -0.13  (0.28)  (0.50) | -0.07  (0.58)  (0.71) | -0.06  (0.61)  (0.71) | -0.05  (0.67)  (0.71) |
| *r_s_* ($\mu$m) | -0.05  (0.66)  (0.71) | 0.25  **(0.03*)**  (0.08) | 0.26  **(0.03*)**  (0.07) | **0.49**  **(<0.001*)**  **(<0.001*)** | **0.35**  **(0.003*)**  **(0.01*)** | 0.07  (0.55)  (0.70) | 0.08  (0.54)  (0.70) | 0.27  **(0.02*)**  (0.06) | 0.07  (0.55)  (0.70) |
| *D_ec_* ($\mu$m^2^/ms) | 0.15  (0.23)  (0.45) | -0.06  (0.61)  (0.71) | -0.11  (0.37)  (0.57) | -0.11  (0.36)  (0.57) | -0.07  (0.59)  (0.71) | 0.13  (0.29)  (0.51) | 0.09  (0.45)  (0.64) | -0.05  (0.65)  (0.71) | 0.003  (0.98)  (0.98) |
| *D_in_* ($\mu$m^2^/ms) | **0.44**  **(<0.001*)**  **(0.002*)** | 0.03  (0.83)  (0.87) | 0.16  (0.18)  (0.36) | 0.19  (0.12)  (0.26) | **0.30**  **(0.01*)**  **(0.047*)** | -0.14  (0.25)  (0.46) | 0.06  (0.64)  (0.71) | 0.08  (0.51)  (0.70) | 0.02  (0.89)  (0.91) |

Data are reported as Pearson’s correlation coefficient with corresponding uncorrected *P*-values (first pair of parentheses) and FDR-corrected *P*-values (second pair of parentheses). * with the bold values denotes statistical significance (*P* < 0.05 or FDR-*P* < 0.05) in Pearson’s correlation coefficient analysis. NODDI = Neurite orientation dispersion and density imaging.

**Supplementary Table 8. Correlations between age and SANDI metrics in the deep gray matter regions**

|  | **Caudate** | **Pallidum** | **Putamen** | **Thalamus** |
| --- | --- | --- | --- | --- |
| *f_is_* | **-0.65**  **(<0.001*)**  **(<0.001*)** | -0.22  (0.07)  (0.10) | **-0.58**  **(<0.001*)**  **(<0.001*)** | -0.25  **(0.04*)**  (0.06) |
| *f_in_* | **0.50**  **(<0.001*)**  **(<0.001*)** | -0.03  (0.81)  (0.85) | **0.49**  **(<0.001*)**  **(<0.001*)** | -0.16  (0.17)  (0.22) |
| *f_ec_* | **0.46**  **(<0.001*)**  **(<0.001*)** | 0.19  (0.10)  (0.14) | **0.27**  **(0.02*)**  **(0.04*)** | **0.42**  **(<0.001*)**  **(<0.001*)** |
| *r_s_* ($\mu$m) | **-0.51**  **(<0.001*)**  **(<0.001*)** | **-0.32**  **(0.006*)**  **(0.01*)** | **-0.62**  **(<0.001*)**  **(<0.001*)** | **-0.32**  **(0.007*)**  **(0.01*)** |
| *D_ec_* ($\mu$m^2^/ms) | **0.50**  **(<0.001*)**  **(<0.001*)** | 0.22  (0.07)  (0.10) | **0.45**  **(<0.001*)**  **(<0.001*)** | **0.31**  **(0.01*)**  **(0.02*)** |
| *D_in_* ($\mu$m^2^/ms) | 0.12  (0.33)  (0.37) | -0.09  (0.48)  (0.52) | -0.02  (0.87)  (0.87) | -0.15  (0.22)  (0.27) |

Data are reported as Pearson’s correlation coefficient with corresponding uncorrected *P*-values (first pair of parentheses) and FDR-corrected *P*-values (second pair of parentheses). * with the bold values denotes statistical significance (*P* < 0.05 or FDR-*P* < 0.05) in Pearson’s correlation coefficient analysis.

**Supplementary Table 9. Correlations between SANDI metrics and normalized volume of each region in the deep gray matter regions**

|  | **Caudate** | **Pallidum** | **Putamen** | **Thalamus** |
| --- | --- | --- | --- | --- |
| *f_is_* | **0.39**  **(<0.001*)**  **(0.003*)** | -0.05  (0.69)  (0.69) | **0.41**  **(<0.001*)**  **(0.002*)** | 0.11  (0.37)  (0.42) |
| *f_in_* | -0.26  **(0.03*)**  (0.05) | 0.16  (0.20)  (0.26) | **-0.34**  **(0.004*)**  **(0.01*)** | **0.40**  **(<0.001*)**  **(0.003*)** |
| *f_ec_* | **-0.30**  **(0.01*)**  **(0.03*)** | -0.14  (0.24)  (0.30) | -0.20  (0.10)  (0.16) | **-0.50**  **(<0.001*)**  **(<0.001*)** |
| *r_s_* ($\mu$m) | **0.55**  **(<0.001*)**  **(<0.001*)** | 0.07  (0.58)  (0.61) | **0.56**  **(<0.001*)**  **(<0.001*)** | 0.21  (0.07)  (0.13) |
| *D_ec_* ($\mu$m^2^/ms) | **-0.33**  **(0.005*)**  **(0.01*)** | -0.07  (0.55)  (0.60) | **-0.36**  **(0.002*)**  **(0.007*)** | **-0.30**  **(0.01*)**  **(0.02*)** |
| *D_in_* ($\mu$m^2^/ms) | 0.16  (0.19)  (0.26) | 0.11  (0.41)  (0.42) | 0.16  (0.19)  (0.26) | **0.28**  **(0.02*)**  **(0.03*)** |

Data are reported as Pearson’s correlation coefficient with corresponding uncorrected *P*-values (first pair of parentheses) and FDR-corrected *P*-values (second pair of parentheses). * with the bold values denotes statistical significance (*P* < 0.05 or FDR-*P* < 0.05) in Pearson’s correlation coefficient analysis.

**
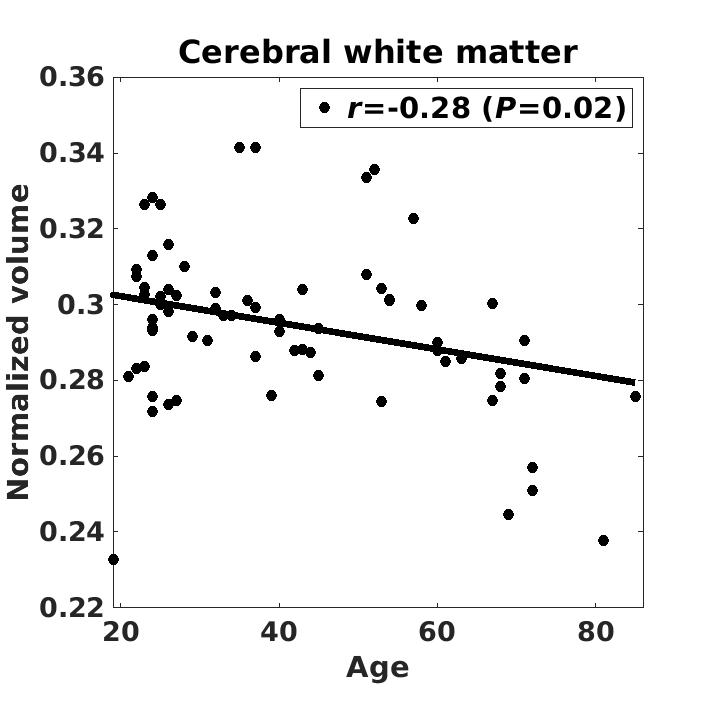
**

**Supplementary Figure 4. Correlations between age and normalized volume of cerebral white matter.** *r* value is Pearson’s correlation coefficient with *P*-value.

**Supplementary Table 10. Correlations between SANDI metrics and age and between SANDI metrics and normalized volume in the cerebral white matter**

|  | *f_is_* | *f_in_* | *f_ec_* | *r_s_* ($\mu$m) | *D_ec_* ($\mu$m^2^/ms) | *D_in_* ($\mu$m^2^/ms) |
| --- | --- | --- | --- | --- | --- | --- |
| **Age** | -0.06  (0.61)  (0.61) | -0.17  (0.16)  (0.27) | 0.25  **(0.03*)**  (0.08) | -0.20  (0.10)  (0.21) | 0.16  (0.19)  (0.29) | -0.11  (0.37)  (0.49) |
| **Normalized volume** | -0.08  (0.49)  (0.58) | **0.51**  **(<0.001*)**  **(<0.001*)** | **-0.32**  **(0.007*)**  **(0.02*)** | **0.48**  **(<0.001*)**  **(<0.001*)** | 0.07  (0.59)  (0.61) | **0.63**  **(<0.001*)**  **(<0.001*)** |

Data are reported as Pearson’s correlation coefficient with corresponding uncorrected *P*-values (first pair of parentheses) and FDR-corrected *P*-values (second pair of parentheses). * with the bold values denotes statistical significance (*P* < 0.05 or FDR-*P* < 0.05) in Pearson’s correlation coefficient analysis.

**Supplementary Table 11. Correlations between age and DTI metrics**

|  | **cerebral WM** | **Entire cerebral**  **cortex** | **Prefrontal** | **Frontal** | **Parietal** | **Temporal** | **Occipital** | **Anterior**  **Cingulate** | **Posterior**  **Cingulate** |
| --- | --- | --- | --- | --- | --- | --- | --- | --- | --- |
| FA | **-0.50**  **(<0.001*)**  **(<0.001*)** | **0.34**  **(0.004*)**  **(0.009*)** | 0.16  (0.18)  (0.20) | **0.46**  **(<0.001*)**  **(<0.001*)** | **0.47**  **(<0.001*)**  **(<0.001*)** | 0.05  (0.66)  (0.68) | 0.24  **(0.04*)**  (0.06) | 0.25  **(0.04*)**  (0.05) | 0.10  (0.40)  (0.43) |
| AD | -0.04  (0.72)  (0.72) | **0.40**  **(<0.001*)**  **(0.002*)** | **0.30**  **(0.01*)**  **(0.02*)** | **0.28**  **(0.02*)**  **(0.03*)** | **0.40**  **(<0.001*)**  **(0.002*)** | **0.27**  **(0.03*)**  **(0.04*)** | 0.09  (0.44)  (0.47) | **0.34**  **(0.004*)**  **(0.009*)** | **0.37**  **(0.002*)**  **(0.004*)** |
| RD | **0.37**  **(0.002*)**  **(0.004*)** | **0.45**  **(<0.001*)**  **(<0.001*)** | **0.30**  **(0.01*)**  **(0.02*)** | 0.20  (0.10)  (0.12) | **0.37**  **(0.001*)**  **(0.004*)** | **0.43**  **(<0.001*)**  **(<0.001*)** | **0.49**  **(<0.001*)**  **(<0.001*)** | **0.26**  **(0.03*)**  **(0.04*)** | **0.37**  **(0.002*)**  **(0.004*)** |
| MD | 0.24  (0.05)  (0.06) | **0.47**  **(<0.001*)**  **(<0.001*)** | **0.31**  **(0.009*)**  **(0.02*)** | 0.23  (0.06)  (0.07) | **0.43**  **(<0.001*)**  **(0.001*)** | **0.40**  **(<0.001*)**  **(0.002*)** | **0.26**  **(0.03*)**  **(0.04*)** | **0.30**  **(0.01*)**  **(0.02*)** | **0.38**  **(0.001*)**  **(0.004*)** |

Data are reported as Pearson’s correlation coefficient with corresponding uncorrected *P*-values (first pair of parentheses) and FDR-corrected *P*-values (second pair of parentheses). * with the bold values denotes statistical significance (*P* < 0.05 or FDR-*P* < 0.05) in Pearson’s correlation coefficient analysis. AD = axial diffusivity; FA = fractional anisotropy; MD = mean diffusivity; RD = radial diffusivity.

**Supplementary Table 12. Correlations between age and DKI metrics**

|  | **Cerebral**  **WM** | **Entire cerebral**  **cortex** | **Prefrontal** | **Frontal** | **Parietal** | **Temporal** | **Occipital** | **Anterior**  **Cingulate** | **Posterior**  **Cingulate** |
| --- | --- | --- | --- | --- | --- | --- | --- | --- | --- |
| FA | **-0.51**  **(<0.001*)**  **(<0.001*)** | 0.14  (0.24)  (0.29) | -0.01  (0.91)  (0.93) | 0.25  **(0.04*)**  (0.05) | **0.32**  **(0.008*)**  **(0.01*)** | -0.14  (0.26)  (0.31) | 0.09  (0.45)  (0.51) | 0.21  (0.08)  (0.10) | 0.01  (0.96)  (0.96) |
| MD | 0.22  (0.07)  (0.09) | **0.48**  **(<0.001*)**  **(<0.001*)** | **0.33**  **(0.005*)**  **(0.01*)** | 0.23  (0.06)  (0.09) | **0.41**  **(<0.001*)**  **(0.002*)** | **0.47**  **(<0.001*)**  **(<0.001*)** | **0.51**  **(<0.001*)**  **(<0.001*)** | **0.29**  **(0.01*)**  **(0.02*)** | **0.39**  **(<0.001*)**  **(0.003*)** |
| AK | **0.31**  **(0.009*)**  **(0.02*)** | **0.44**  **(<0.001*)**  **(<0.001*)** | **0.49**  **(<0.001*)**  **(<0.001*)** | **0.38**  **(0.001*)**  **(0.003*)** | 0.22  (0.07)  (0.09) | **0.50**  **(<0.001*)**  **(<0.001*)** | -0.04  (0.75)  (0.79) | **0.53**  **(<0.001*)**  **(<0.001*)** | 0.25  **(0.04*)**  (0.05) |
| RK | -0.20  (0.10)  (0.12) | **0.37**  **(0.002*)**  **(0.004*)** | **0.35**  **(0.003*)**  **(0.007*)** | **0.32**  **(0.007*)**  **(0.01*)** | **0.27**  **(0.02*)**  **(0.04*)** | **0.36**  **(0.002*)**  **(0.005*)** | 0.07  (0.59)  (0.65) | **0.38**  **(0.001*)**  **(0.003*)** | **0.36**  **(0.003*)**  **(0.005*)** |
| MK | -0.05  (0.69)  (0.74) | **0.48**  **(<0.001*)**  **(<0.001*)** | **0.44**  **(<0.001*)**  **(<0.001*)** | **0.37**  **(0.002*)**  **(0.004*)** | **0.31**  **(0.009*)**  **(0.02*)** | **0.49**  **(<0.001*)**  **(<0.001*)** | 0.09  (0.45)  (0.51) | **0.47**  **(<0.001*)**  **(<0.001*)** | **0.37**  **(0.002*)**  **(0.004*)** |

Data are reported as Pearson’s correlation coefficient with corresponding uncorrected *P*-values (first pair of parentheses) and FDR-corrected *P*-values (second pair of parentheses). * with the bold values denotes statistical significance (*P* < 0.05 or FDR-*P* < 0.05) in Pearson’s correlation coefficient analysis. AD = axial diffusivity; AK = axial kurtosis; FA = fractional anisotropy; MK = mean kurtosis; RK = radial kurtosis.

**Supplementary Table 13. correlations between DTI metrics and normalized volume**

|  | **Cerebral WM** | **Entire cerebral**  **cortex** | **Prefrontal** | **Frontal** | **Parietal** | **Temporal** | **Occipital** | **Anterior**  **Cingulate** | **Posterior**  **Cingulate** |
| --- | --- | --- | --- | --- | --- | --- | --- | --- | --- |
| FA | -0.18  (0.14)  (0.30) | -0.11  (0.35)  (0.46) | -0.06  (0.59)  (0.66) | -0.22  (0.07)  (0.20) | -0.21  (0.08)  (0.20) | 0.10  (0.41)  (0.50) | -0.17  (0.17)  (0.34) | -0.22  (0.07)  (0.20) | -0.14  (0.25)  (0.41) |
| AD | -0.21  (0.08)  (0.20) | -0.25  **(0.04*)**  (0.18) | -0.21  (0.08)  (0.20) | -0.16  (0.20)  (0.36) | -0.24  **(0.04*)**  (0.18) | -0.04  (0.71)  (0.78) | 0.04  (0.75)  (0.78) | -0.16  (0.18)  (0.34) | -0.13  (0.27)  (0.42) |
| RD | -0.02  (0.90)  (0.90) | -0.33  **(0.006*)**  (0.14) | -0.27  **(0.02*)**  (0.18) | -0.13  (0.28)  (0.42) | -0.23  (0.05)  (0.20) | -0.27  **(0.03*)**  (0.18) | -0.18  (0.13)  (0.28) | -0.08  (0.52)  (0.61) | -0.12  (0.34)  (0.46) |
| MD | -0.10  (0.42)  (0.51) | -0.32  **(0.008*)**  (0.14) | -0.25  **(0.04*)**  (0.18) | -0.14  (0.24)  (0.41) | -0.26  **(0.03*)**  (0.18) | -0.19  (0.11)  (0.27) | -0.04  (0.76)  (0.78) | -0.11  (0.36)  (0.46) | -0.13  (0.30)  (0.43) |

Data are reported as Pearson’s correlation coefficient with corresponding uncorrected *P*-values (first pair of parentheses) and FDR-corrected *P*-values (second pair of parentheses). * with the bold values denotes statistical significance (*P* < 0.05 or FDR-*P* < 0.05) in Pearson’s correlation coefficient analysis. AD = axial diffusivity; FA = fractional anisotropy; MD = mean diffusivity; RD = radial diffusivity.

**Supplementary Table 14. Correlations between DKI metrics and normalized volume**

|  | **Cerebral WM** | **Entire cerebral**  **cortex** | **Prefrontal** | **Frontal** | **Parietal** | **Temporal** | **Occipital** | **Anterior**  **Cingulate** | **Posterior**  **Cingulate** |
| --- | --- | --- | --- | --- | --- | --- | --- | --- | --- |
| FA | 0.14  (0.24)  (0.33) | 0.08  (0.50)  (0.56) | 0.14  (0.26)  (0.36) | 0.04  (0.73)  (0.77) | -0.05  (0.66)  (0.72) | 0.22  (0.07)  (0.11) | -0.13  (0.30)  (0.38) | -0.26  **(0.03*)**  (0.06) | -0.16  (0.19)  (0.28) |
| MD | -0.22  (0.07)  (0.11) | **-0.33**  **(0.006*)**  **(0.03*)** | **-0.30**  **(0.01*)**  **(0.04*)** | -0.12  (0.32)  (0.39) | -0.24  **(0.0493*)**  (0.09) | **-0.28**  **(0.02*)**  **(0.049*)** | -0.18  (0.14)  (0.21) | -0.10  (0.39)  (0.45) | -0.12  (0.31)  (0.38) |
| AK | 0.32  **(0.007*)**  **(0.03*)** | **-0.43**  **(<0.001*)**  **(0.003*)** | **-0.44**  **(<0.001*)**  **(0.003*)** | **-0.30**  **(0.01*)**  **(0.04*)** | -0.27  **(0.02*)**  (0.06) | **-0.44**  **(<0.001*)**  **(0.003*)** | 0.10  (0.39)  (0.45) | **-0.38**  **(0.001*)**  **(0.009*)** | **-0.28**  **(0.02*)**  **(0.04*)** |
| RK | 0.35  **(0.003*)**  **(0.02*)** | **-0.28**  **(0.02*)**  **(0.04*)** | **-0.31**  **(0.009*)**  **(0.03*)** | -0.23  (0.06)  (0.11) | -0.24  **(0.046*)**  (0.09) | -0.22  (0.06)  (0.11) | -0.03  (0.76)  (0.78) | -0.21  (0.09)  (0.13) | -0.05  (0.69)  (0.74) |
| MK | 0.40  **(<0.001*)**  **(0.006*)** | **-0.39**  **(<0.001*)**  **(0.007*)** | **-0.38**  **(0.001*)**  **(0.009*)** | -0.26  **(0.03*)**  (0.07) | **-0.29**  **(0.01*)**  **(0.04*)** | **-0.35**  **(0.003*)**  **(0.02*)** | -0.04  (0.84)  (0.84) | **-0.29**  **(0.01*)**  **(0.04*)** | -0.12  (0.31)  (0.38) |

Data are reported as Pearson’s correlation coefficient with corresponding uncorrected *P*-values (first pair of parentheses) and FDR-corrected *P*-values (second pair of parentheses). * with the bold values denotes statistical significance (*P* < 0.05 or FDR-*P* < 0.05) in Pearson’s correlation coefficient analysis. AD = axial diffusivity; AK = axial kurtosis; FA = fractional anisotropy; MK = mean kurtosis; RK = radial kurtosis.

**
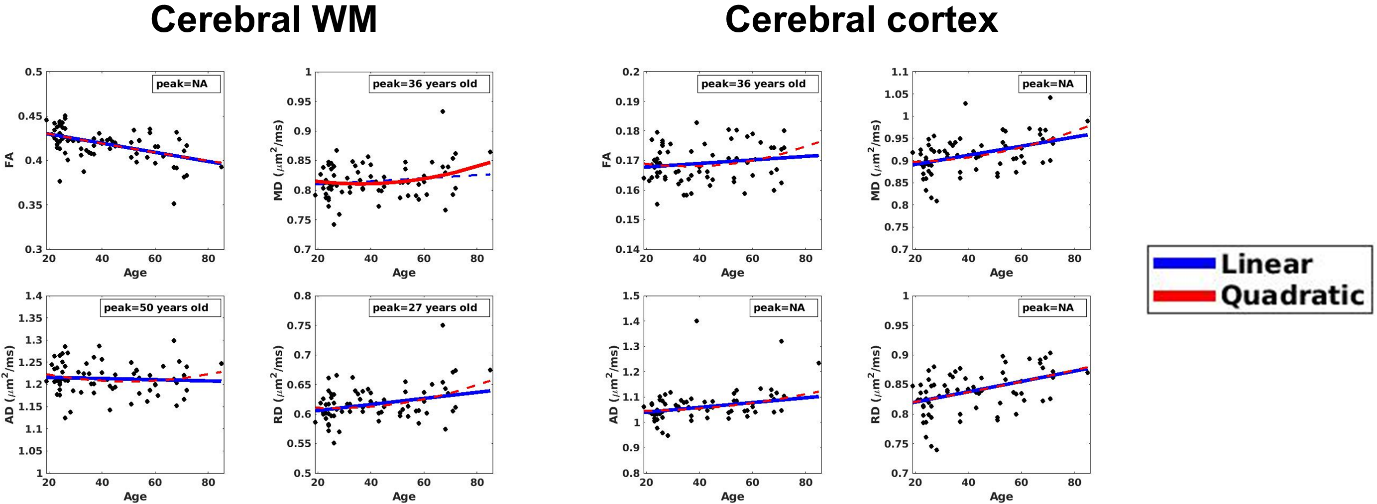
**

**Supplementary Figure 5. Linear (blue) and quadratic (red) regression analyses of DTI metrics with age.** The peak age for quadratic regression is indicated. The regression line with the higher R-squared value is depicted as a thicker, solid line. NA = not applicable.

**Supplementary Table 15. Goodness of fit assessed by R-squared value for the DTI metrics with respect to age**

|  | **FA** | **MD** | **AD** | **RD** |
| --- | --- | --- | --- | --- |
| **Cerebral WM** |  |  |  |  |
| Linear | 0.341 | 0.099 | -0.081 | 0.260 |
| Quadratic | 0.321 | 0.123 | -0.083 | 0.254 |
| **Cerebral Cortex** |  |  |  |  |
| Linear | 0.609 | 0.261 | 0.651 | 0.147 |
| Quadratic | 0.608 | 0.255 | 0.615 | 0.134 |

**
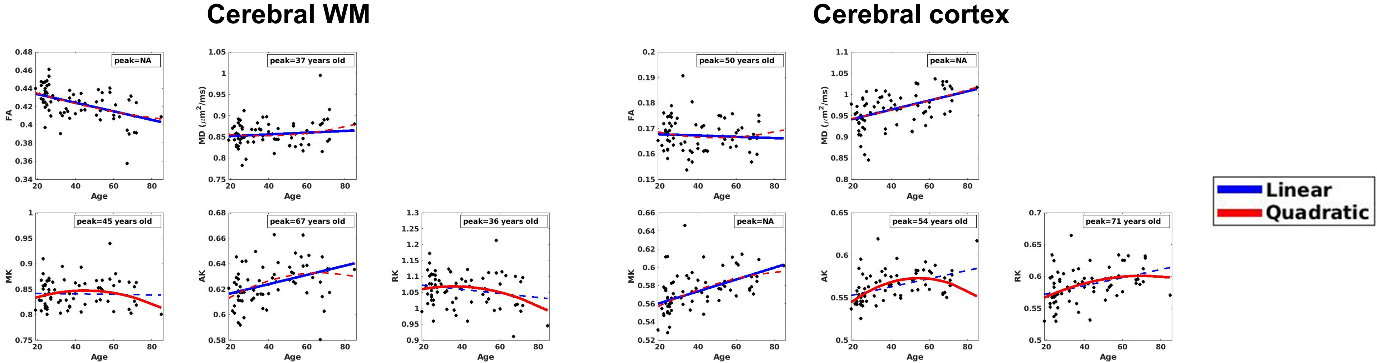
**

**Supplementary Figure 6. Linear (blue) and quadratic (red) regression analyses of DKI metrics with age.** The peak age for quadratic regression is indicated. The regression line with the higher R-squared value is depicted as a thicker, solid line. NA = not applicable.

**Supplementary Table 16. Goodness of fit assessed by R-squared value for the DKI metrics with respect to age**

|  | **FA** | **MD** | **AK** | **RK** | **MK** |
| --- | --- | --- | --- | --- | --- |
| **Cerebral WM** |  |  |  |  |  |
| Linear | 0.283 | 0.203 | 0.209 | 0.001 | 0.051 |
| Quadratic | 0.272 | 0.196 | 0.183 | 0.018 | 0.068 |
| **Cerebral Cortex** |  |  |  |  |  |
| Linear | 0.390 | 0.187 | 0.241 | 0.125 | 0.364 |
| Quadratic | 0.387 | 0.170 | 0.350 | 0.130 | 0.352 |

**
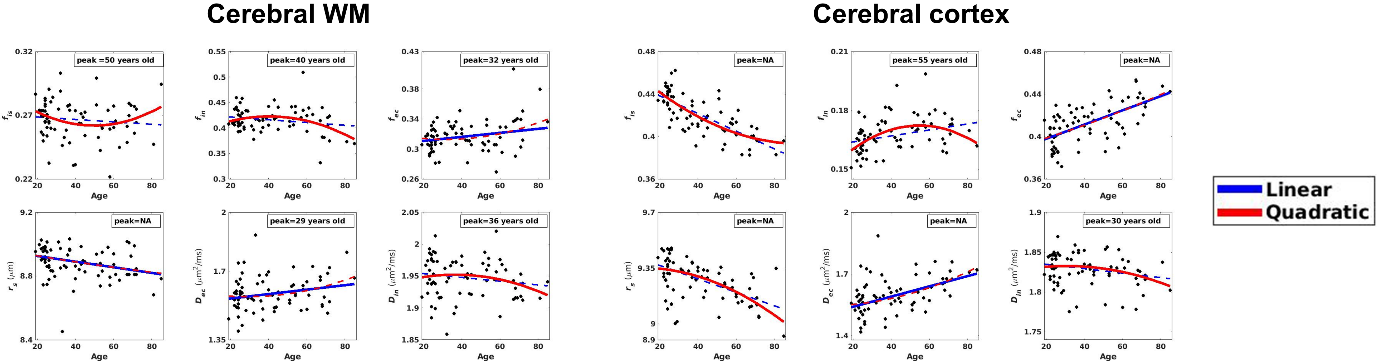
**

**Supplementary Figure 7. Linear (blue) and quadratic (red) regression analyses of SANDI metrics with age.** The peak age for quadratic regression is indicated. The regression line with the higher R-squared value is depicted as a thicker, solid line. NA = not applicable.

**Supplementary Table 17. Goodness of fit assessed by R-squared value for the SANDI metrics with respect to age**

|  | ***f_is_*** | ***f_in_*** | ***f_ec_*** | ***r_s_* (**$\boldsymbol{\mu}$**m)** | ***D_ec_* (**$\boldsymbol{\mu}$**m^2^/ms)** | ***D_in_* (**$\boldsymbol{\mu}$**m^2^/ms)** |
| --- | --- | --- | --- | --- | --- | --- |
| **Cerebral WM** |  |  |  |  |  |  |
| Linear | 0.031 | 0.203 | 0.257 | 0.226 | 0.064 | -0.023 |
| Quadratic | 0.039 | 0.265 | 0.234 | 0.198 | 0.055 | -0.021 |
| **Cerebral Cortex** |  |  |  |  |  |  |
| Linear | 0.519 | 0.149 | 0.292 | 0.330 | 0.318 | 0.050 |
| Quadratic | 0.532 | 0.233 | 0.272 | 0.349 | 0.312 | 0.063 |


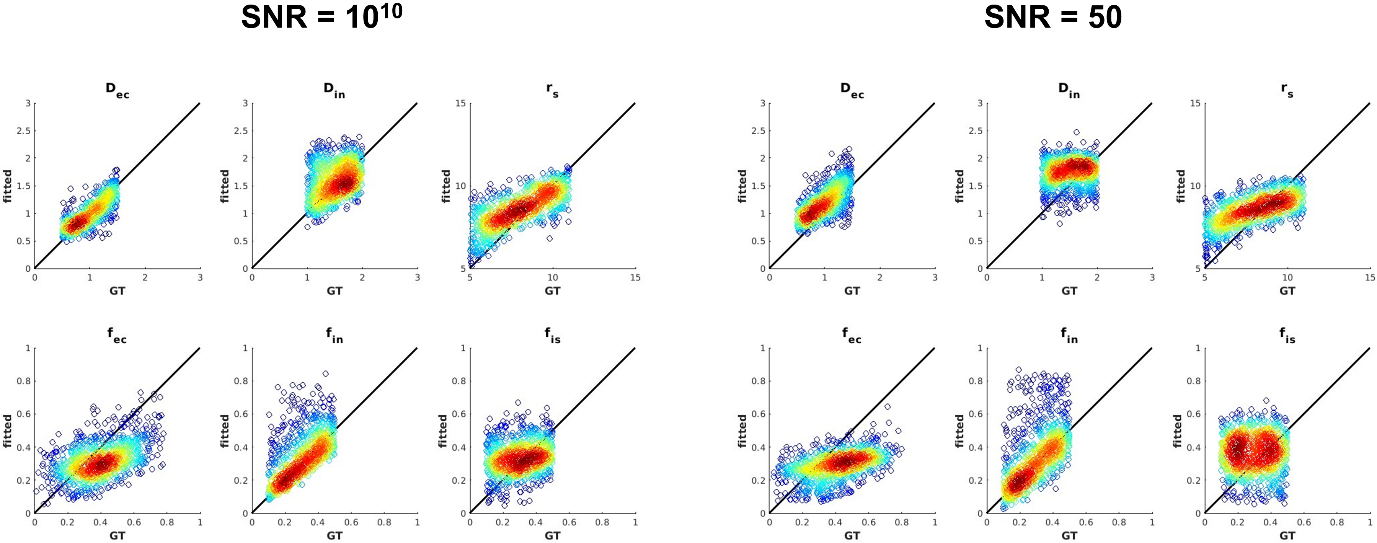


**Supplementary Figure 8. Noise propagation with randomly distributed parameters in the SANDI fitting using random forest regression algorithm.** GT represents ground truth value and fitted represents the fitted value from SANDI fitting.
